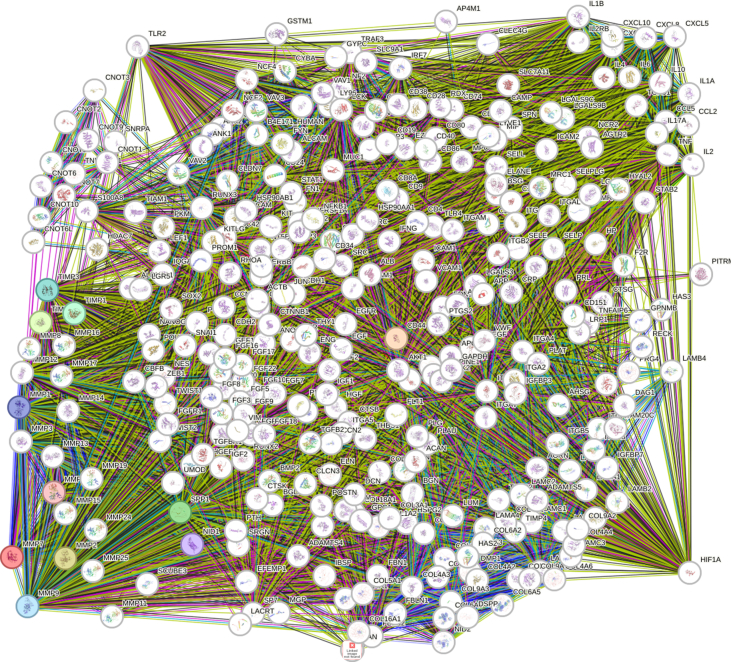# Matrix metalloproteinase/Fas ligand (MMP/FasL) interaction dynamics in COVID-19: An *in silico* study and neuroimmune perspective

**DOI:** 10.1016/j.heliyon.2024.e30898

**Published:** 2024-05-15

**Authors:** Kiarash Saleki, Cena Aram, Parsa Alijanizadeh, Mohammad Hossein Khanmirzaei, Zahra Vaziri, Mohammad Ramzankhah, Abbas Azadmehr

**Affiliations:** a Student Research Committee, Babol University of Medical Sciences, Babol, Iran; b Department of e-Learning, Virtual School of Medical Education and Management, Shahid Beheshti University of Medical Sciences(SBMU), Tehran, Iran; c USERN Office, Babol University of Medical Sciences, Babol, Iran; d National Elite Foundation, Mazandaran Province Branch, Mazandaran, Iran; e Department of Cell & Molecular Biology, Faculty of Biological Sciences, Kharazmi University, Tehran, Iran; f School of Medicine, Tehran University of Medical Sciences, Babol, Iran; g Universal Scientific Education and Research Network (USERN), Tehran, Iran; h Department of Immunology, Babol University of Medical Sciences, Babol, Iran

**Keywords:** Matrix metalloproteinase, Fasa ligand (FasL), Hyperinflammation, COVID-19, Immunoinformatics

## Abstract

**Background:**

The initiator of cytokine storm in Coronavirus disease (COVID-19) is still unknown. We recently suggested a complex interaction of matrix metalloproteinases (MMPs), Fas ligand (FasL), and viral entry factors could be responsible for the cytokine outrage In COVID-19. We explored the molecular dynamics of FasL/MMP7-9 in COVID-19 conditions *in silico* and provide neuroimmune insights for future.

**Methods:**

We enrolled and analyzed a clinical cohort of COVID-19 patients, and recorded their blood Na + levels and temperature at admission. A blood-like molecular dynamics simulation (MDS) box was then built. Four conditions were studied; MMP7/FasL (healthy), MMP7/FasL (COVID-19), MMP9-FasL (healthy), and MMP9/FasL (COVID-19). MDS was performed by GROningen MAchine for Chemical Simulation (GROMACS). We analyzed bonds, short-range energies, and free binding energies to draw conclusions on the interaction of MMP7/MMP9 and FasL to gain insights into COVID-19 immunopathology. Genevestigator was used study RNA-seq/microarray expression data of MMPs in the cells of immune and nervous systems. Finally, epitopes of MMP/FasL complexes were identified as drug targets by machine learning (ML) tools.

**Results:**

MMP7-FasL (Healthy), MMP7-FasL (COVID-19), MMP9-FasL (Healthy), and MMP9-FasL (COVID-19) systems showed 0, 1, 4, and 2 salt bridges, indicating MMP9 had more salt bridges. Moreover, in both COVID-19 and normal conditions, the number of interacting residues and surface area was higher for MMP9 compared to MMP7 group. The COVID-19 MMP9-FasL group had more H-bonds compared to MMP7-FasL group (12 vs. 7). 15 epitopes for FasL-MMP9 and 10 epitopes for FasL-MMP7 were detected. Extended MD simulation for 100 ns confirmed stronger binding of MMP9 based on Molecular Mechanics Generalized Borne Surface analysis (MM-GBSA) and Coul and Leonard-Jones (LJ) short-range energies.

**Conclusions:**

MMP9 interacts stronger than MMP7 with FasL, however, both molecules maintained strong interaction through the MDS. We suggested epitopes for MMP-FasL complexes as valuable therapeutic targets in COVID-19. These data could be utilized in future immune drug and protein design and repurposing efforts.

## Highlights

• MMP9 interacts stronger than MMP7 with FasL, however, both molecules maintained strong interaction through the MDS.
• We suggested epitopes for MMP-FasL complexes as valuable therapeutic targets in COVID-19.
• The COVID-19 MMP9-FasL group had more H-bonds compared to MMP7-FasL group.
• Interaction of MMPs and FasL may have diverse neuroimmune implications in COVID-19.
• Epitopes from MMP-FasL could be a valuable target for drug design efforts.

## 1 Introduction

The coronavirus disease-2019 (COVID-19) pandemic is still ongoing, despite development of efficient vaccines. The continued emergence of new variants emphasizes the need to explore the immunopathological underpinnings of severe acute respiratory syndrome coronavirus-2 (SARS-CoV-2) [1,2].

Hyperinflammation is implicated in COVID-19 neuroimmunopathology and its involvement of respiratory and central nervous systems. Critical COVID-19 patient had significantly higher levels of pro-inflammatory cytokines, in particular IL-1β, IL-6, IL-8, tumor necrosis factor (TNF-α), as well as IFN-γ [2]. However, detecting the starter of the upregulation in the cytokine network requires further investigation.

We hypothesized that Fas/FasL signaling could be an initiator mechanism regulating cytokine storm and neuroimmune involvement by interacting with SARS-CoV-2 entry elements (neuropilin-1 (NRP-1) and cytokine storm [3]. A recent study by Saleki et al. found that membrane-bound (m)Fas/mFasL and soluble (s)Fas/sFasL were distinctly mediated in COVID-19 patients with different severity levels. *In silico* interactions were also analyzed by molecular dynamics simulation (MDS) in COVID-19 conditions such as hyponatremia and fever. sFasL was found to be a severity and mortality marker in COVID-19 patients with robust diagnostic performance. Also, sFasL was associated with lung injury in COVID-19 patients [4]. Another study also showed than attenuated sFasL resulted in TNF-driven neutrophil necroptosis in critical COVID-19 patients [5].

One of COVID-19 mechanisms that is involved in regulation of Fas/FasL signaling is matrix metalloproteinase (MMP)-mediated cleaved of mFasL to sFasL. MMP levels are elevated in COVID-19 patients. MMP-8 and MMP-2 cooperate in COVID-19 immunopathological lung damage by oxidative stress [6]. MMP-7 is a major marker that can detect COVID-19 cases requiring invasive mechanical ventilation [7]. Additionally, MMP-7 has been shown to cleave a number of bioactive substrates that could accelerate fibrosis, like osteopontin [8,9]. MMPs are able to break-down excreted proteins like collagen and elastin fibers of the extracellular matrix (ECM) [10], are necessary for ECM restructuring, wound recovery, and vessel formation, and a number of them, such as MMP-1 and MMP-9, have been found to play a role in the immunopathogenesis of interstitial pulmonary fibrosis (IPF) [[11], [12], [13], [14]], a condition which is associated with COVID-19 [15] and other conditions [16]. MMP-7 synthesis is raised in clinical IPF and preclinical models for lung fibrosis, whereas MMP-7 knockout rodents display diminished fibrotic reactions [11,17]. Damages associated with the cytokine storm are the disequilibrium of the MMPs/TIMPs ratio, increase deposition of fragments generated by extracellular matrix disruption, and substantial fibrin deposition. These events may lead to pulmonary endothelial damage, several diseases (such as pulmonary edema and acute respiratory distress syndrome) and organ failure, which may result in death [18].

FasL cleavage into sFasL is mediated through metalloproteinases like MMP-3, MMP-7, and MMP-9 [7]. These MMPs could be inhibited by various molecules. For instance, tissue inhibitors of metalloproteinases (TIMPs) denote endogenous proteins that mediate MMPs [19]. It is therefore important to investigate the interaction of different MMPs with FasL to provide insights for optimal targeting of MMPs, and the role of change in the body fluid environments their molecular interactions. Here, we study the molecular interactions of various MMPs with Fas/FasL to identify differences in molecular affinity and changes in interaction in COVID-19-like conditions (fever, electrolyte changes). Also, we discuss neuroimmune insights and hypotheses on the role of MMPs and FasL in neuropathological involvements by SARS-CoV2. The objective of this study is to evaluate interactions of MMP7/MMP9 with FasL and suggest therapeutic targets against COVID-19. The wide range of MMP protein-protein interactions and possible pathways, such as our neuroimmune hypothesis, linking MMP to novel molecules such as FasL, as well as to multi-organ damage involving CNS and lungs. This underscores the value of unraveling the dynamics of MMPs in the context of SARS-CoV2 infection. The results of this study could be utilized to design candidates that target MMPs, in order to mediate hyperinflammation, and attenuate COVID-19 neuroimmunopathology.

## 2 Methods

### 2.1 Modelling of MMP and FasL proteins

3D structure of FasL (PDB ID: 4MSV), MMP7 (PDB ID: 7WXX), and MMP9 (PDB ID: 1L6J) were retrieved from the protein databank (PDB). Single-template modelling by UCSF MODELLER was used to fill in the missing residues for all proteins. MODELLER is a robust tool for homology or comparative modeling of protein 3D construct. By aligning sequence to be modeled (target) to the sequence of known structures (templates). Next, the program computes a desired number of possible constructs comprising non-hydrogen elements. MODELLER employs comparative modeling by meeting of spatial restraints [20]. Loop refinement was used to improve the energy of loops with high energies.

### 2.2 Protein quality validation

The utilization of torsion angles to explain polypeptide and protein conformation was developed in the research team of G.N. Ramachandran, and after half a century still is a major tool for protein structure evaluation. The φ/ψ plot of the amino acid residues in a peptide is called the Ramachandran plot. The plot utilizes φ quantities on the x-axis and the ψ quantities on the y-axis to forecast the probable protein structure. The angle range for both axes is −180° to +180° [21]. ProSA is another approach for evaluating tertiary structures of proteins for possible errors. ProSA is used to explore errors in experimentally produced models, theoretical constructs and protein design. ProSA (prosa.services.came.sbg.ac.at) uses z-score to verify structure against experimental constructs with the same number of residues. The overall quality score calculated by ProSA for a special input construct is shown in a graph that indicates the scores of every experimentally quantified protein structure accessible in the Protein Data Bank (PDB) [22]. ERRAT ProCheck is another tool that scores amino acids in 9-residue intervals [4,[23], [24], [25], [26]]. The quality of the modeled protein was verified by Ramachandran plot, ProSA, and UCLA SAVES 6 analyses (Supplementary material).

### 2.3 Docking study of MMPs and FasL

PatchDock is a geometry-reliant molecular docking tool. The algorithm detects docking transformations that produce robust spatial complementarity. These transformations induce either wide interface regions or small levels of steric clashes. A wide interface is ensured to comprise various matched local features of the matched elements with complementary features. This tool categorizes the Connolly dot surface representation of the elements into concave, convex and flat pats. Afterward, complementary parts are joined to produce transformations. Each candidate transformation is evaluated by an internal scoring that considers both geometric fitness and energy of solvation. Ultimately, an RMSD clustering is applied to each structure to reduce redundant structures [27]. PatchDock server was used to dock FasL with MMP7 and MMP9. The best docking solution was selected and taken to the molecular dynamics simulation (MDS) step.

### 2.4 Molecular dynamics simulation of MMPs and FasL in healthy and COVID-19-like conditions

To validate the docking study and further evaluate interactions of MMP7/9 with FasL, we employed MDS by GROningen MAchine for Chemical Simulations (GROMACS). GROMACS package is a well-established and fast software for MDS studies [28]. After creating a topology using a high-precision all-atom forcefield OPLS/AA, we set up our simulation system by creating a PBC triclinic box around the docked MMP7/9-FasL complex. The box was then filled with TIP3P water. Solvent molecules were randomly replaced with Na^+^ and Cl^−^ ions to neutralize the system and reach blood-like osmolarities. The system was minimized using the steepest descent algorithm to reach the maximum force <1000.0 kJ mol^−1^ nm^−1^. Then, the system underwent two NVT and NPT equilibration steps to reach 310 K and 0 bar, respectively. Hyponatremia and fever are important findings in COVID-19 patients. For fever and hyponatremia conditions, we chose mild fever that is often found in COVID-19 patients (38.85 °C) and 130 Na^+^ levels, in line with our recent study findings [3,4,29]. Finally, four separate MD production runs were performed for 10 ns allowing the system to evolve. After centering the protein using *gmx trjconv*, root mean square deviation (RMSD), root-mean-square fluctuation (RMSF), and gyration were analyzed by *gmx rms, gmx rmsf*, and *gmx gyrate* tools.

### 2.5 Analysis of MMP/FasL interactions

Two kinds of short-range potential were computed: Lennard–Jones short-range (LJ-SR) and Coulombic short-range (Coul-SR) potential. We computed the overall sum of LJ-SR and Coul-SR potential between MMPs and FasL. As SR energies do not capture the total binding energies, Molecular mechanics with generalized Born and surface area solvation (MM/GBSA) were also calculated to give a better view of binding free energies (cadd.zju.edu.cn/hawkdock/) [30]. Finally, PDBSum generate server (ebi.ac.uk/thornton-srv/databases/pdbsum/Generate.html) was used to plot the interacting residues, number of H-bonds, salt bridges, contact areas, pores, and clefts [31] in the MMP/9-FasL complexes in both healthy and disease conditions. Data were analyzed for MMP7 compared to MMP9 and healthy compared to COVID-19 conditions.

### 2.6 B- and T-cell immune epitope prediction

Immune epitopes are valuable tools for targeting by immunotherapeutic approaches. We predicted B-cell and T-cell epitopes of MMP7/9-FasL complexes in COVID-19 using the sequence and 3D PDB data. DiscoTope is a method for predicting discontinuous epitopes from 3D structures of proteins in PDB format (see Reference at the end of the tutorial). For B-cell epitopes, Discotope v1.1 and ElliPro tools (Immune Epitopes Database (IEDB)) with default settings were employed. Also, CTLPred and HTLPred servers were recruited to detect T-cell epitopes based on their sequence. Top candidates are introduced as robust targets for therapy.

### 2.7 STRING protein-protein interaction of MMPs

We performed STRING protein-protein interactions of MMPs (https://string-db.org/) to identify related regulatory pathways and proteins. In this study, STRING protein-protein interactions (PPIs) of MMPs were analyzed both for a small number of proteins and an extended number of proteins. Moreover, STRING is a database of known and forecasted PPIs. The reactions include direct (physical) and non-direct (functional) associations; Assessments are based on Bioinformatics prediction, from information transfer among organisms, and from reactions aggregated from the rest of (primary) repositories (string-db.org/).

### 2.8 Microarray analysis of condition-specific and tissue-specific MMP7/9 expression

We employed Genevestigator (Nebion, Switzerland) to evaluate condition-specific and tissue-specific expression of MMP7/9. Data selection was HS_AFFY_U133PLUS_2-0. Condition-specific analyses were performed. Cut-off for *p* value was set to p < 0.001. Fold changes were plotted at |values| > 3. 6674 perturbations were analyzed from the selected data. 254 anatomical parts were also analyzed for tissue-specific expression analysis. Values were sorted in descending order for all analyses.

## 3 Results

### 3.1 Structure modeling

Experimental structures of proteins in this study have missing residues (Supplementary Material). First, non-standard residues were removed from structure. We used UCSF MODELLER to perform template-based modeling and fill missing residues in the experimental structures. MODELLER produced 5 models, for FasL, MMP7, and MMP9. For FasL, molpdf, DOPE score, and GA341 were found 1014.33, −11265.56, and 1.00, respectively. For MMP7, molpdf, DOPE score, and GA341 were found 965.59, −17894.57, and 1.00, respectively. Finally, for MMP9, molpdf, DOPE score, and GA341 were found 3016.68, −44830.47, and 1.00, respectively. A GA341 over 0.6 indicates good models and lower DOPE scores confirms favorable energy profile of the modeled protein [76,77]. The structures were prepared for docking by UCSF Chimera DockPrep.

### 3.2 Docking of MMPs/FasL

PatchDock server was used (RMSD clustering 4.0) to dock MMP7 and MMP9 with FasL. For both MMP7-FasL and MMP9-FasL complexes, the top 20 solutions were sorted by docking score (Table 1, Table 2). The highest ranking solution for MMP7 and MMP9 showed a docking score, surface, ACE, and transformation of 13470, 1909.20, −396.67, [−0.89 0.53 0.84–12.76 19.06–28.86]; 19478, 2728.30, −66.58, [−3.06 0.31 -0.04 -65.14 47.52 -0.49]. The docking score was higher for MMP9 compared to MMP7 for binding with FasL; however, as this needs verification in blood-like situations and special COVID-19-like conditions, the best solution was taken to the Molecular dynamics simulation (MDS) step. After MDS, contact residues were analyzed by Chimera for COVID-19 conditions (Supplementary material).

**Table 1:** MMP7-FasL docking solutions.

Solution No	Score	Area	ACE	Transformation
1	13470	1909.2	−396.67	−0.89 0.53 0.84–12.76 19.06–28.86
2	13464	1723.3	319.75	2.50 1.02 2.58–23.27 -27.26 27.53
3	13098	1659.9	−81.07	−1.23 0.89 2.92–63.01 -2.68 -19.81
4	13044	2008	−44.32	−1.23 0.89 2.92–63.01 -2.68 -19.81
5	12934	2248.3	−444.15	−0.87 0.94 2.47–55.30 7.01–17.04
6	12920	1680.3	380.82	−1.71 -0.33 -2.85 -31.14–3.58 -64.25
7	12804	1835	−145.43	0.91 -0.05 -2.73 -54.80–29.36 -29.08
8	12802	1846.9	−386.82	−0.48 0.13 0.79–17.49 23.82 -43.74
9	12762	1785.6	−253.6	2.28 -0.38 -2.97 -48.64–0.87 -30.11
10	12756	1735.3	−259.71	−0.46 0.58 0.62–13.40 17.75 -27.46
11	12706	1992.3	367.04	−1.42 -0.84 -2.87 -30.68–3.33 -65.86
12	12640	1910.5	190	−1.96 -0.90 -3.02 -38.87 0.59–61.30
13	12478	1869.3	−24.89	2.26 0.53 1.92–31.37 16.86 -8.89
14	12408	1632	478.45	1.95 0.45 2.11–30.47 -18.4113.70
15	12380	1547.8	115.91	2.99 0.65 2.31-42.07 -1.93 -9.83
16	12376	1852.7	−183.12	−2.33 -0.71 -1.12 -28.28–22.83 -53.04
17	12360	1616.1	402.05	2.32 0.97 2.44–26.60 -27.48 25.94
18	12330	1748.3	336.42	−1.40 0.46 0.54 4.17–22.25 -1.02
19	12258	1497.2	464.71	−0.95 -0.87 2.94 -22.66 1.52–83.14
20	12238	1815.2	44.40	2.04 0.02–0.59 17.19–20.72 -27.89

**Table 2:** MMP9-FasL docking solutions.

Solution No	Score	Area	ACE	Transformation
1	19478	2728.30	−66.58	−3.06 0.31 -0.04 -65.14 47.52 -0.49
2	18358	2909.70	−38.01	1.23 -0.09 2.39 -15.06 -39.80–98.29
3	17794	2736.80	150.04	−1.65 0.07 0.19–67.43 -36.27 -2.77
4	17794	2481.60	23.05	2.93 0.39 -0.35 -31.73 68.41 -22.75
5	16146	2237.20	373.71	−0.36 0.86 0.2.19–56.16 44.04–93.34
6	15864	2028.20	27.46	2.73 -0.77 0.73–85.50 -4.66 37.38
7	15702	2753.50	95.46	−2.99 -0.73 0.20–35.42 27.17 35.05
8	15486	2294.40	338.13	−1.56 -0.38 -0.11 -57.12–66.60 29.13
9	15340	1951.80	−124.41	2.02 -1.00 -1.96 32.94 -15.42 -35.44
10	15240	2161.60	25.97	−2.36 0.73 2.98 32.39–58.83 15.19
11	14982	2446.60	237.06	2.99 -0.70 0.94 -79.31 -9.81 45.48
12	14982	2894.00	−68.56	2.08 -0.78 1.78 -52.53 -82.16 7.76
13	14956	2033.70	210.31	−1.75 0.02 0.31–65.79 -36.65 -3.03
14	14862	2321.40	19.39	−2.07 0.39 0.85–28.23 -54.74 -21.90
15	14774	1934.10	199.05	−2.67 0.08–2.13 41.18 8.95 -6. 17
16	14728	2568.60	239.15	−1.71 0.19 0.43–66.70 -45.33 -7.15
17	14658	1971.30	−8.32	−3.13 0.54 -0.32 -46.52 62.98 -15.95
18	14602	2158.10	34.49	−2.19 0.21 1.63–29.43 -75.58 3.48
19	14556	1723.50	194.25	−1.78 -0.21 0.32–55.93 -33.02 8.05
20	14548	1996.00	−116.64	−0.96 -0.87 2.15 47.11 22.76 38.30

error estimates.

### 3.3 Molecular dynamics simulation of MMP/FasL

The best solution of the docked MMP7-FasL and MMP9-FasL was selected to build blood-like conditions resembling healthy patients and special fever and hyponatremia COVID-19-like conditions. A triclinic MD box was built with Periodic Boundary Conditions (PBC). The box was solvated with TIP3P water, which is a variation based on CHARMM (MacKerell) denotes a 3-area fixed water element with charges and Lennard-Jones (LJ) features considered for all 3 atoms [32]. Ions were added to reach blood-like concentrations similar to healthy patients and COVID-19 (hyponatremia variant). Then, all four MMP-FasL systems were minimized using the steepest descent algorithm. All systems converged to F_max_ < 1000. MMP7 (Healthy, COVID-19) and MMP9 (Healthy, COVID-19) converged to the target of minimization in 644 steps Potential (Energy = −7.6310362e+05), 1014 steps (Energy = −7.7362425e+05); 1910 steps.

(Energy = −1.2311611e+06), and 1494 steps (Energy = −1.2201881e+06), respectively. In the same order, density of systems was found 1039.49 (0.91), 1037.64 (0.83), 1045.49 (0.44), and 1044.92 (0.52). Minimized systems were taken through two equilibration systems, namely NVT and NPT each for 100 ps, to reach the temperature targets (COVID-19 fever, healthy) and pressure of 0 bar, as described in methods. Finally, MD run was performed for all four systems of MMP7-FasL (Healthy), MMP7-FasL (COVID-19), MMP9-FasL (Healthy), and MMP9-FasL (COVID-19) systems (Fig. 1). Analysis of root mean square deviation (RMSD), root mean square fluctuation (RMSD), and gyration was performed. Results showed RMSD stabilized after 5 ns for all systems. Visualization of the MD trajectory as well as RMSD and gyration analyses confirmed that the molecules did not expand in both conditions (Fig. 2A–H). Ultimately, the stabilized final frames were extracted from the MD trajectory files.

**Fig. 1:**
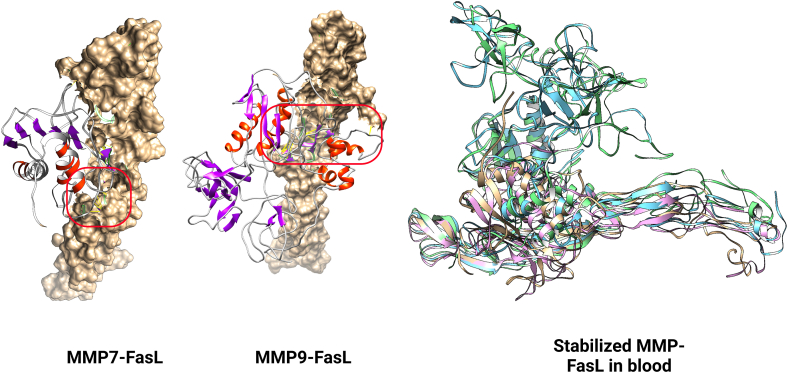
MMPs and FasL docking study.

**Fig. 2:**
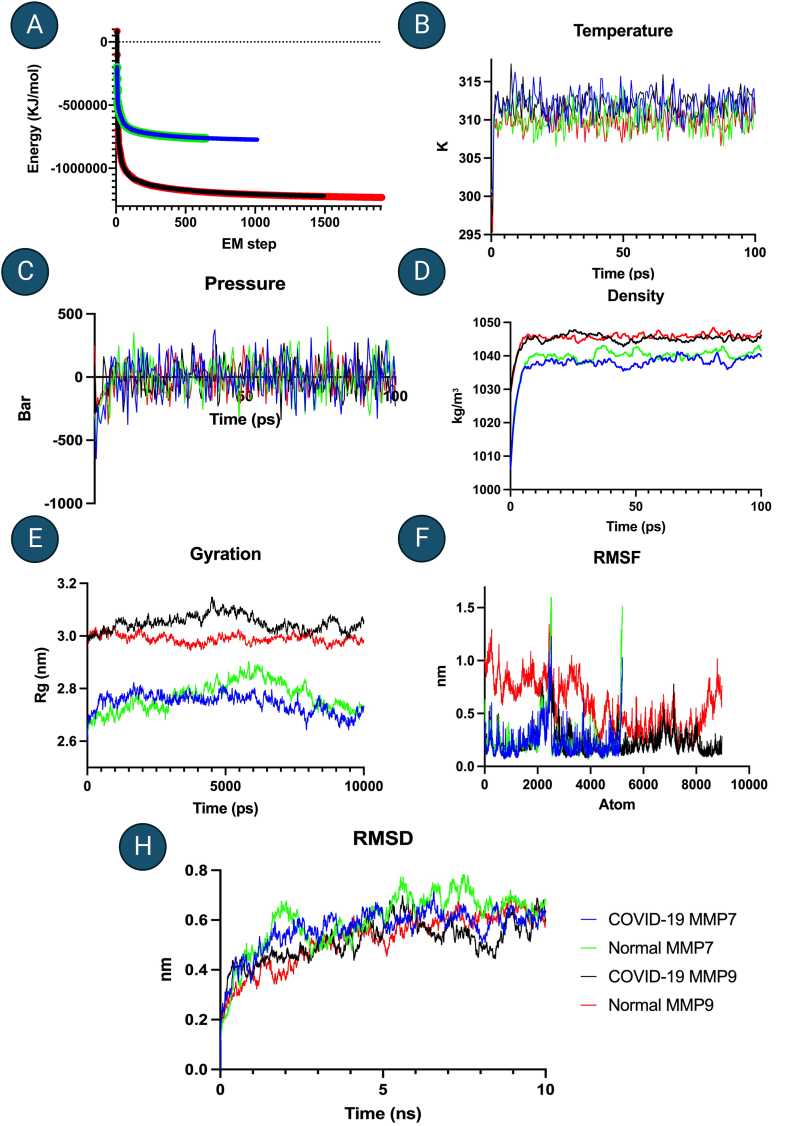
Molecular dynamics simulation of MMP-FasL MDS of MMP-FasL complexes in COVID-19 are depicted. Steps shown include A: minimization, B: NVT equilibration-temperature, C: NPT equilibration-pressure, D: NPT equilibration-density, E: Final MD run-Gyration, F: Final MD run RMSF, G/H: Final MD run RMSD for all groups.

### 3.4 Interactions of MMP/FasL in healthy and COVID-19-like conditions

We investigated interactions of MMP7/9-FasL based on three domains for all simulated systems. Various molecular bonds (H-bond, salt bridges, disulphide bonds) short range energies (Leonard Jones (LJ) and Columbic (Coul)), and free-binding energies by molecular mechanics with generalized Born and surface area solvation (MM/GBSA).

**Fig. 3a:**
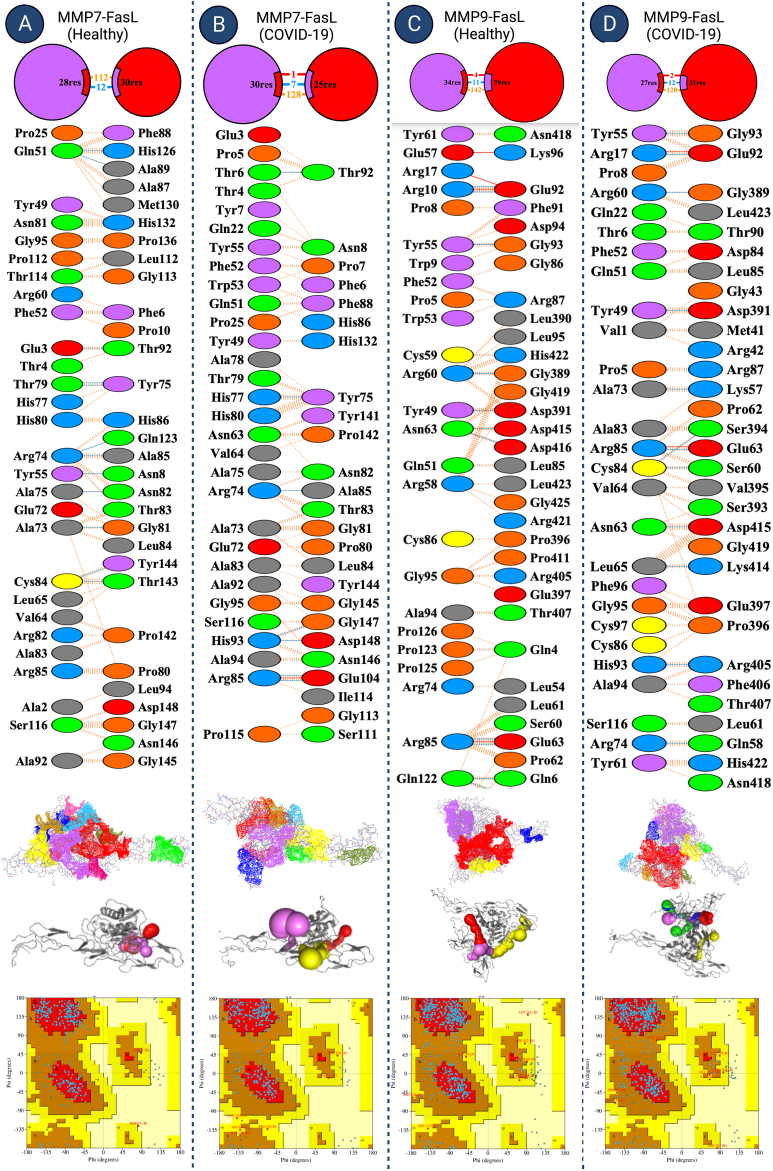

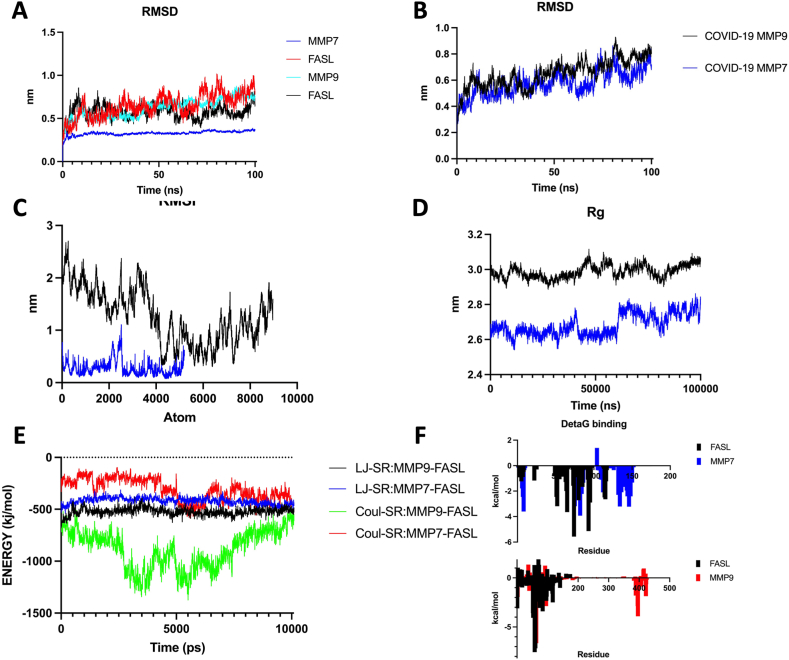
MMP/FasL interactions analysis Various bonds, amino acids, and protein quality analyses are shown. Fig. 3b A-F. Extended Molecular Dynamics Simulation Analysis of MMP7/9-FasL complex

**Fig. 4:**
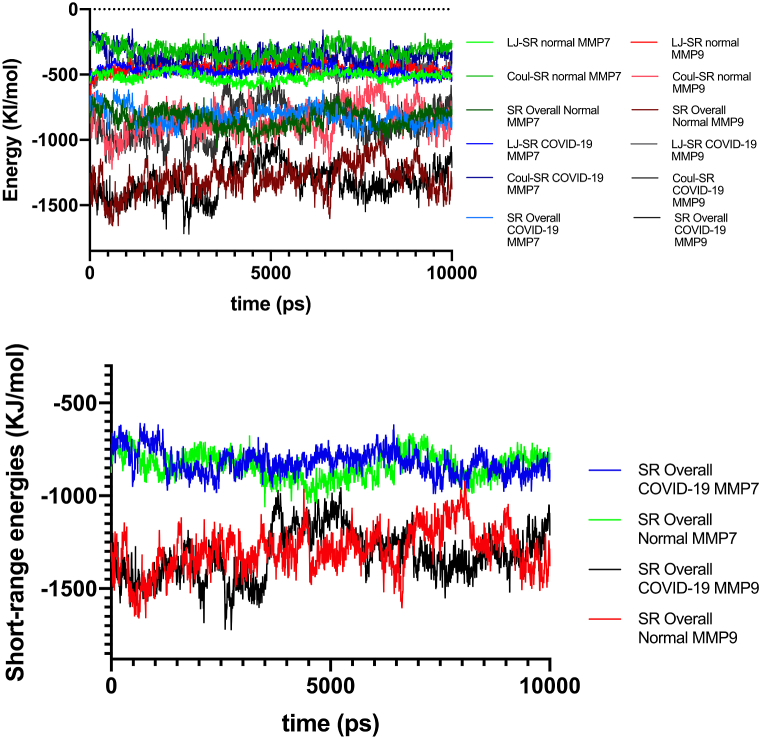
**Short-range** energy analyses of MMP/FasL.

MMP7-FasL (Healthy), MMP7-FasL (COVID-19), MMP9-FasL (Healthy), and MMP9-FasL (COVID-19) systems showed 0, 1, 4, and 2 salt bridges, indicating MMP9 had more salt bridges. Overall, in both COVID-19 and normal conditions, the number of interacting residues and surface area was higher for MMP9 compared to MMP7 group. The COVID-19 MMP9-FasL group had more H-bonds compared to MMP7-FasL group (12 vs. 7). Bonds, non-bonded contact interacting residues of MMP7/9-FasL for all simulations have been demonstrated (Fig. 3A A-D, Table 3). Short-range (SR) energies were also analyzed. MMP9-FasL (COVID-19) showed the most negative (lowest) Coul-SR (−863.6) and overall SR energies (−1317.9) compared to all other groups. Coul-SR energies were generally lower in COVID-19 vs. healthy groups for both MMP7 and MMP9 (Table 4a). Extended analysis of MDS trajectory for 100 ns re-confirmed that the binding of MMP9 was stronger as demonstrated by most energies calculated in MMGBSA analysis (Table 4b, Fig. 3b A-F). Analysis of SR energies by MD time showed that any group of MMP9 had lower overall SR energies compared to any group of MMP7 almost consistently. However, a comparison of healthy vs. COVID-19 groups for MMP7/9 did not show a sustained difference. The final stabilized frames of simulations indicated a slightly lower overall SR in COVID-19 conditions (Fig. 4). MM-GBSA indicated strong interactions for both MMP7 and MMP9 conditions. Similar to the previous analysis, free binding energy was more favorable (negative) for MMP9 groups compared to the MMP7 group. We suggested that interactions of MMP-FasL for each person with COVID-19 may be personalized based on their blood profile and other factors, however, in the specific settings of our study which were derived from a real clinical sample of clinical COVID-19 patients, there was a strong interaction in the case of mild fever and hyponatremia which are commonly found in COVID-19 patients. The interactions may generally be stronger for MMP9 compared to MMP7 (Table 5). The stabilized frames of MMP-FasL complexes were selected to predict targets for immune-mediating therapies and *in silico* protein design.

**Table 3:** Bonds in MMP-FasL interactions.

Complex	Condition	No. of interface residues	Interface area (Å2)	No. of salt bridges	No. of disulphide bonds	No. of hydrogen bonds	No. of non-bonded contacts
MMP7-FasL	*Normal*	28	1439	–	–	12	112
		30	1446				
	*COVID-19*	30	1316	1	–	7	128
		25	1396				
MMP9-FasL	*Normal*	34	1792	4	–	11	142
		39	1752				
	*COVID-19*	27	1500	2	–	12	120
		31	1443

**Table 4a:** MM-GBSA analysis of MMP-FasL.

Energy components (kcal/mol)	COVID-19 MMP7	Normal MMP7	COVID-19 MMP9	Normal MMP9
	**Last frame of MD**			
Binding free energy	−81.44	−81.14	−92.09	−92.4
Van der Wall energy (vdw)	−148.6	−150.03	−146.34	−160.14
Electrostatic energy	−139.51	−110.86	−852.03	−873.12
Electrostatic solvation energy (GB)	222.77	197.25	924.72	962.02
Non-electrostatic solvation energy (SA)	−16.1	−17.49	−18.44	−21.16

**Table 4b:** MMGBSA analysis of extended MDS trajectory of MMP-FasL complex.

Energy components (kcal/mol)	COVID-19 MMP7	COVID-19 MMP9
	**Average Energies**	
Binding free energy	−75.59	−92.09
Van der Wall energy (vdw)	−128.11	−146.34
Electrostatic energy	−156.13	−852.03
Electrostatic solvation energy (GB)	224.47	924.72
Non-electrostatic solvation energy (SA)	−15.81	−18.44

**Table 5:** Average short-range energies of MMP7-/MMP9- FasL.

Short-range energies	MMP9	MMP7
Healthy	LJ-SR: 446.8 [12]	LJ-SR: 518.8 (9.7)
	Coul-SR: 846.5 [23]	Coul-SR: 318.5 [11]
COVID-19	LJ-SR: 454.3 [7]	LJ-SR: 471.4 (8.3)
	Coul-SR: 863.6 [38]	Coul-SR: 347.6 (9.4)

*Note:* Values in parentheses represent error estimates.

### 3.5 Immune epitope mapping for FasL and MMP7/9

Immune epitopes were predicted from the MMP7-FasL and MMP9-FasL complexes by the IEDB tool. Both B-cell (continuous, discontinuous) and T-cell (HTL, CTL) epitopes were evaluated based on the sequence and PDB structure of the proteins. 15 epitopes for FasL-MMP9 and 10 epitopes for FasL-MMP7 were detected. Discontinuous epitopes were also plotted. Sequence range and percentile rank of epitopes have been provided (Table 6a, Table 6b, Table 6c, Table 6da-d and Fig. 5) (see Table 7a, Table 7ba,b).

**Table 6a:** Linear B-cell epitopes. Chain A: FASL Chain B: MMP9.

No	Chain	Start	End	Peptide	Number of residues	Score
1	A	121	174	CQPCPPGTFSASSSSSEQCQPHRNCTALGLALNVPGSSSHDTLCTSTGHHHHHH	54	0.886
2	A	1	62	VAETPTYPWRDAETGERLVCAQCPPGTFVQRPCRRDSPTTCGPCPPRHYTQFWNYLERCRYC	62	0.767
3	A	101	110	ASCPPGAGVI	10	0.635
4	A	75	80	ACHATH	6	0.572
5	B	292	311	YRWCATTANYDRDKLFGFCP	20	0.776
6	B	226	236	TTDGRSDGLPW	11	0.726
7	B	356	365	TTSNFDSDKK	10	0.721
8	B	239	290	TTANYDTDDRFGFCPSERLYTRDGNADGKPCQFPFIFQGQSYSACTTDGRSD	52	0.704
9	B	202	208	RFGNADG	7	0.678
10	B	36	51	TRVAEMRGESKSLGPA	16	0.648
11	B	328	341	CVFPFTFLGKEYST	14	0.631
12	B	1	16	APRQRQSTLVLFPGDL	16	0.624
13	B	421	425	RHLYG	5	0.592
14	B	89	103	QTFEGDLKWHHHNIT	15	0.539
15	B	129	145	WSAVTPLTFTRVYSRDA	17	0.525

**Table 6b:** Discontinuous B-cell epitopes.

No	Residues	Number of residues	Score
1	A:A101, A:S102, A:C103, A:P104, A:P105, A:G106, A:A107, A:G108, A:V109, A:I110, A:T119, A:*C*121, A:Q122, A:P123, A:C124, A:P125, A:P126, A:G127, A:T128, A:F129, A:S130, A:A131, A:S132, A:S133, A:S134, A:S135, A:S136, A:E137, A:Q138, A:C139, A:Q140, A:P141, A:H142, A:R143, A:N144, A:C145, A:T146, A:A147, A:L148, A:G149, A:L150, A:A151, A:L152, A:N153, A:V154, A:P155, A:G156, A:S157, A:S158, A:S159, A:H160, A:D161, A:T162, A:L163, A:C164, A:T165, A:S166, A:T167, A:G168, A:H169, A:H170, A:H171, A:H172, A:H173, A:H174	65	0.84
2	A:V1, A:A2, A:E3	3	0.828
3	B:A1, B:P2, B:Q4	3	0.754
4	B:R202, B:F203, B:G204, B:N205, B:A206, B:D207, B:G208, B:F217, B:E218, B:G219, B:R220, B:C225, B:T227, B:D228, B:G229, B:R230, B:S231, B:D232, B:G233, B:L234, B:P235, B:W236, B:T239, B:T240, B:A241, B:N242, B:Y243, B:D244, B:T245, B:D246, B:D247, B:R248, B:F249, B:G250, B:F251, B:C252, B:P253, B:S254, B:E255, B:R256, B:L257, B:Y258, B:T259, B:D261, B:G262, B:N263, B:A264, B:D265, B:G266, B:K267, B:P268, B:C269, B:Q270, B:F271, B:P272, B:F273, B:I274, B:G277, B:Q278, B:S279, B:Y280, B:S281, B:A282, B:C283, B:T284, B:T285, B:D286, B:G287, B:R288, B:S289, B:D290, B:G291, B:Y292, B:R293, B:W294, B:C295, B:A296, B:T297, B:T298, B:A299, B:N300, B:D302, B:R303, B:D304, B:K305, B:L306, B:F307, B:G308, B:F309, B:C310, B:P311, B:R313	92	0.708
5	A:T4, A:P5, A:T6, A:Y7, A:P8, A:W9, A:R10, A:D11, A:A12, A:E13, A:T14, A:G15, A:E16, A:R17, A:L18, A:V19, A:C20, A:A21, A:Q22, A:C23, A:P24, A:P25, A:G26, A:T27, A:F28, A:V29, A:Q30, A:R31, A:P32, A:C33, A:R34, A:R35, A:D36, A:S37, A:P38, A:T39, A:T40, A:C41, A:G42, A:P43, A:C44, A:P45, A:P46, A:R47, A:H48, A:Y49, A:T50, A:W53, A:N54, A:Y55, A:L56, A:C59, A:R60, A:Y61, A:C62, A:N63, A:A75, A:C76, A:H77, A:A78, A:T79, A:H80, A:N81, A:R82, B:R87, B:T90, B:E92, B:G93, B:D94, B:L95, B:K96, B:W97, B:H98, B:H99, B:H100, B:A127, B:S130, B:A131, B:V132, B:T133, B:P134, B:L135, B:T136, B:R421, B:L423, B:Y424, B:G425	87	0.694
6	B:T226, B:S323, B:V329, B:F330, B:P331, B:F332, B:T333, B:F334, B:L335, B:G336, B:K337, B:E338, B:Y339, B:S340, B:T341, B:T343, B:T356, B:T357, B:S358, B:N359, B:D361, B:S362, B:D363, B:K365	24	0.649
7	B:T36, B:V38, B:A39, B:E40, B:R42, B:G43, B:E44, B:S45, B:K46, B:S47, B:L48, B:G49, B:P50, B:A51, B:T64, B:G65, B:E66, B:L67	18	0.594
8	B:R3, B:R5, B:Q6, B:S7, B:T8, B:L9, B:V10, B:L11, B:F12, B:P13, B:G14, B:D15, B:L16, B:R17, B:N322, B:E345, B:G346, B:R347	18	0.549

**Table 6c:** Linear B-cell epitopes A: FASL B: MMP7.

No	Chain	Start	End	Peptide	Number of residues	Score
1	A	138	174	QCQPHRNCTALGLALNVPGSSSHDTLCTSTGHHHHHH	37	
2	A	1	49	VAETPTYPWRDAETGERLVCAQCPPGTFVQRPCRRDSPTTCGPCPPRHY	49	0.767
3	A	119	133	TQCQPCPPGTFSASS	15	0.635
4	A	99	110	EHASCPPGAGVI	12	0.572
5	A	55	62	YLERCRYC	8	0.776
6	B	163	175	QKLYGKRSNSRKK	13	0.726
7	B	45	63	GKEIPLHFRKVVWGTADIM	19	0.721
8	B	12	25	WTSKVVTYRIVSYT	14	0.704
9	B	28	31	LPHI	4	0.678
10	B	68	79	RGAHGDSYPFDG	12	0.648

**Table 6d:** Discontinuous epitopes.

No	Residues	Number of residues	Score
1	A:R10, A:D11, A:A12, A:E13, A:T14, A:G15, A:E16, A:R17, A:R35	9	0.877
2	B:Y19, B:K54, B:V56, B:W57	4	0.81
3	A:A101, A:S102, A:C103, A:P104, A:P105, A:G106, A:A107, A:G108, A:I110, A:Q120, A:*C*121, A:Q122, A:P123, A:C124, A:P125, A:P126, A:G127, A:T128, A:F129, A:S130, A:A131, A:S132, A:S133, A:S134, A:E137, A:Q138, A:C139, A:Q140, A:P141, A:H142, A:R143, A:N144, A:C145, A:T146, A:A147, A:L148, A:G149, A:L150, A:A151, A:L152, A:N153, A:V154, A:P155, A:G156, A:S157, A:S158, A:S159, A:H160, A:D161, A:T162, A:L163, A:C164, A:T165, A:S166, A:T167, A:G168, A:H169, A:H170, A:H171, A:H172, A:H173, A:H174	62	0.774
4	B:M1, B:G2, B:Y3, B:W12, B:S14, B:K15, B:V16, B:V17, B:T18, B:R20, B:I21, B:V22, B:S23, B:Y24, B:S38, B:N42, B:G45, B:K46, B:E47, B:I48, B:P49, B:L50, B:H51, B:F52, B:R53, B:V55, B:G58, B:T59, B:A60, B:D61, B:M63, B:S133, B:S134, B:D135, B:P136, B:N137, B:A138, B:L154, B:S155, B:Q156, B:D157, B:I159, B:K160, B:Q163, B:Y166, B:G167, B:K168, B:R169, B:S170, B:N171, B:S172, B:K174, B:K175	32	0.654
5	B:L28, B:P29, B:H30, B:I31, B:D34, B:R35	6	0.633
6	A:V1, A:A2, A:E3, A:T4, A:P5, A:T6, A:Y7, A:P8, A:W9, A:L18, A:V19, A:C20, A:A21, A:Q22, A:C23, A:P24, A:P25, A:G26, A:T27, A:F28, A:V29, A:Q30, A:R31, A:P32, A:C33, A:R34, A:D36, A:S37, A:P38, A:T39, A:T40, A:C41, A:G42, A:P43, A:C44, A:P45, A:P46, A:R47, A:H48, A:T50, A:N54, A:Y55, A:L56, A:E57, A:R58, A:C59, A:R60, A:Y61, A:C62, A:N63, A:C76, A:H77, A:A78, A:H80, B:T92, B:G93	56	0.604

**Fig. 5:**
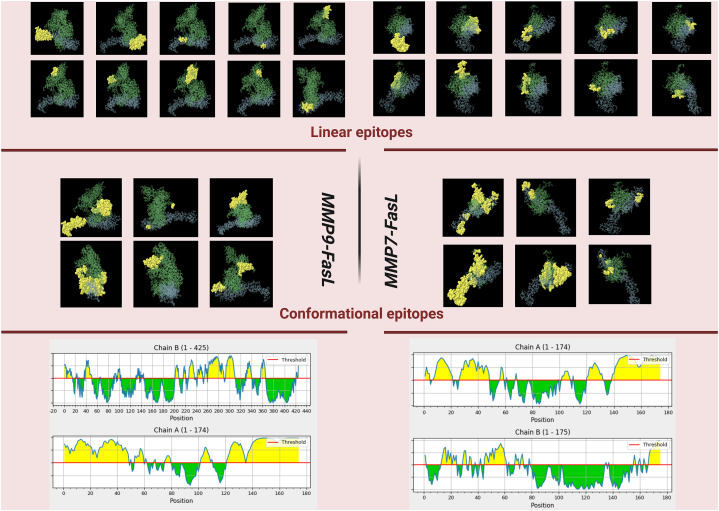
Epitope prediction.

**Table 7a:** T-cell epitope prediction for FasL.

	Epitope	MHC I Alleles	Start	End	Percentile rank
FasL CTL/HTL	QPHRNCTAL	HLA-B*07:02	140	148	0.04
	HYTQFWNYL	HLA-A*24:02	48	56	0.04
	VAETPTYPW	HLA-B*58:01	1	9	0.11
	RACHATHNR	HLA-A*31:01	74	82	0.08
	**Epitope**	**MHC II Alleles**	**Start**	**End**	**Percentile rank**
	ALGLALNVPGSSSHD	HLA-DRB3*02:02	147	161	0.92

**Table 7b:** T-cell epitope prediction for MMP7/9.

	Epitope	MHC I Alleles	Start	End	Percentile rank
MMP7 CTL/HTL	SSDPNAVMY	HLA-A*01:01	133	141	0.01
	YSLFPNSPKW	HLA-B*57:01	3	12	0.01
	FLYAATHQL	HLA-A*02:01	116	124	0.01
	LVSKALNMW	HLA-B*57:01	36	44	0.04
	**Epitope**	**MHC II Alleles**	**Start**	**End**	**Percentile rank**
	GINFLYAATHQLGHS	HLA-DRB1*07:01	113	127	0.11
	IVSYTRDLPHITVDR	HLA-DRB3*02:02	21	35	1.4
MMP9 CTL/HTL	**Epitope**	**MHC I Alleles**	**Start**	**End**	**Percentile rank**
	APRQRQSTL	HLA-B*07:02	1	9	0.01
	QTFEGDLKW	HLA-B*58:01	89	97	0.01
	TTDGRSDGY	HLA-A*01:01	284	292	0.01
	**Epitope**	**MHC II Alleles**	**Start**	**End**	**Percentile rank**
	YPMYRFTEGPPLHKD	HLA-DRB1*07:01	401	415	0.06

### 3.6 STRING protein-protein interactions analysis of MMPs

Analysis of MMPs PPIs showed related regulatory pathways. Network nodes showing protein splice isoforms or post-translational alterations are collapsed, and each node represents all the proteins generated via a single, protein-coding gene locus. Colored nodes indicate query proteins and the first line of interactor molecules. Where a 3D PDB structure is available it is provided in a filled node. Known (based on curated databanks, experimentally verified), predicted (based on gene neighborhood, gene fusions, and gene co-occurrence), and other (based on co-expression, homology, text mining, and interactions) were plotted in different colors (supplementary materials). An 11-node analysis showed that the average number of node degrees was 8.55, the number of edges was 47, the average local clustering coefficient was 0.904, and the PPI enrichment *p-value* was 1.64e-12 (Fig. 6). Moreover, an extended 331-node analysis showed the average number of node degrees was 83.5, the number of edges was 13820, the average local clustering coefficient was 0.658, and the PPI enrichment *p-value* was 1.0e-16 (Fig. 7). Predicted functional partners of MMP7 included MMP1, MMP2, MMP9, MMP10, TIMP1, TIMP2, TIMP3, SPP1, CD44, and NID1.

**Fig. 6:**
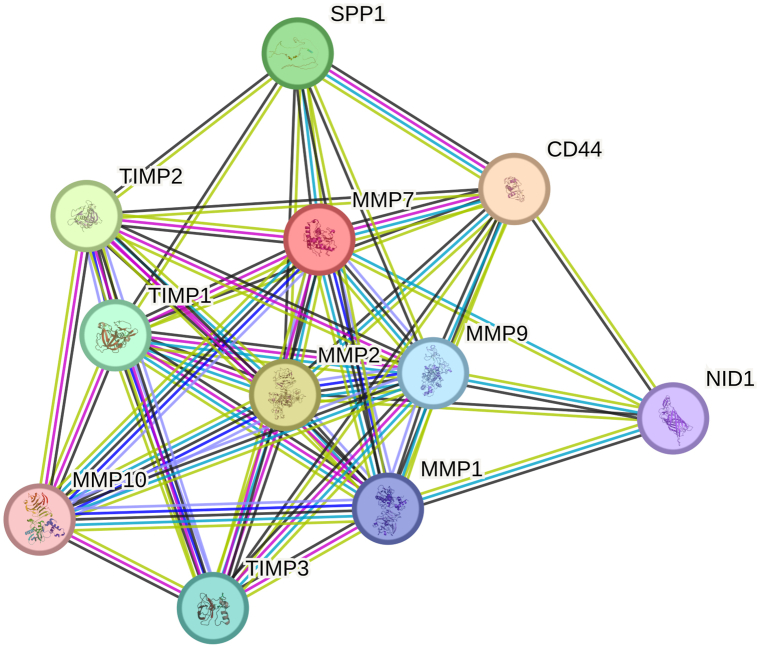
STRING protein-protein interactions of MMPs.

**Fig. 7:**
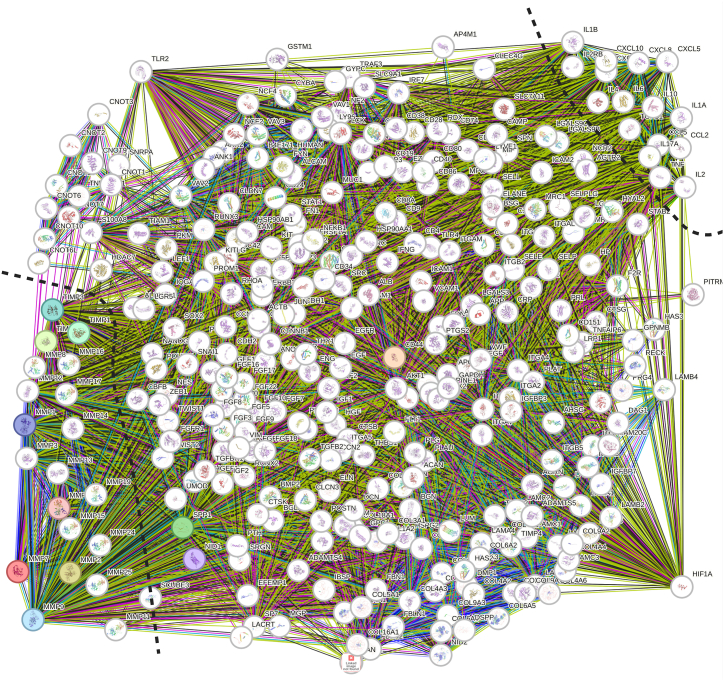
Extended STRING protein-protein interactions of MMPs.

### 3.7 Microarray condition-specific and tissue-specific MMP7/9 expression analysis

Condition-specific analysis of MMP7 showed highest values in ovarian tumor study, colorectal adenoma study, and transwell differentiation study. Several pancreatic islet studies showed the lowest expression among analyzed data. Fold changes ranged from −212.76 to 119.01. For MMP9, Langerhans cell histiocytosis, conditioned medium study, and CML study showed highest values. The B-CLL study showed lowest values. Fold changes ranged from −39.18 to 283.83. It should be noted that these analyses indicate that MMP7/9 expression is dysregulated in many respiratory conditions (Fig. 1, Fig. 2 Supp). The tissue-specific study revealed boosted expression of MMP7/9 in the nasal epithelium, bronchial epithelium, and small airway. Moreover, identifying at-risk populations and key targets of the multi-organ damage by SARS-CoV2 are important topics. In this regard, these clues regarding expression levels of MMPs may be beneficial to estimate the involvement of MMP pathways in various diseases or tissues.

Moreover, we reported data for specific CNS cells. We plotted Microarray/RNAseq analyses for CNS, respiratory, and immune cells by Genevestigator. Also, differential expression of MMP7/9, FAS, FASLG, ACE2, and NRP1 was plotted describing expression specific CNS cell types and immune cells, providing better neuroimmune context for results provided in the present study (Fig. 8, Fig. 9, Fig. 10 A-F).

**Fig. 8:**
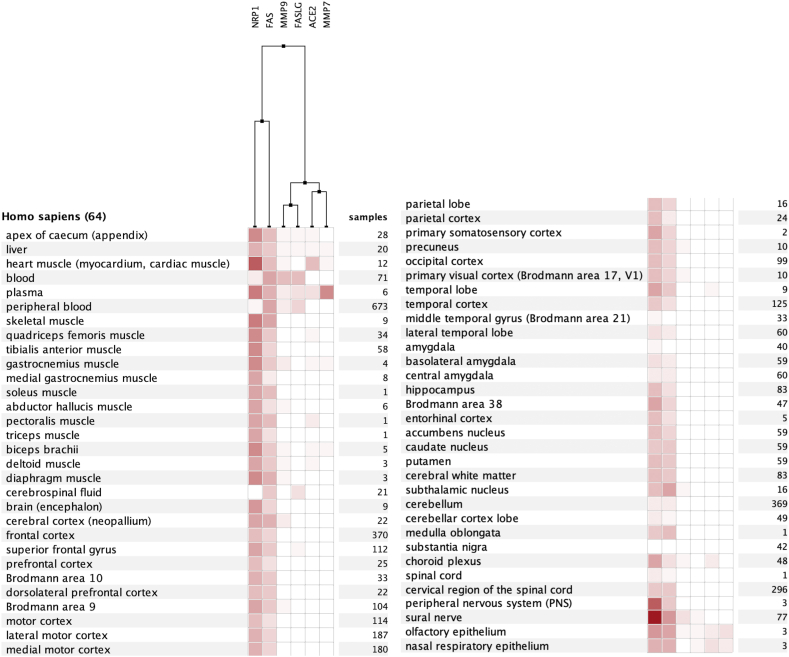
Heatmap expression of MMPs, FasL, and related molecules in specific CNS cell types.

**Fig. 9:**
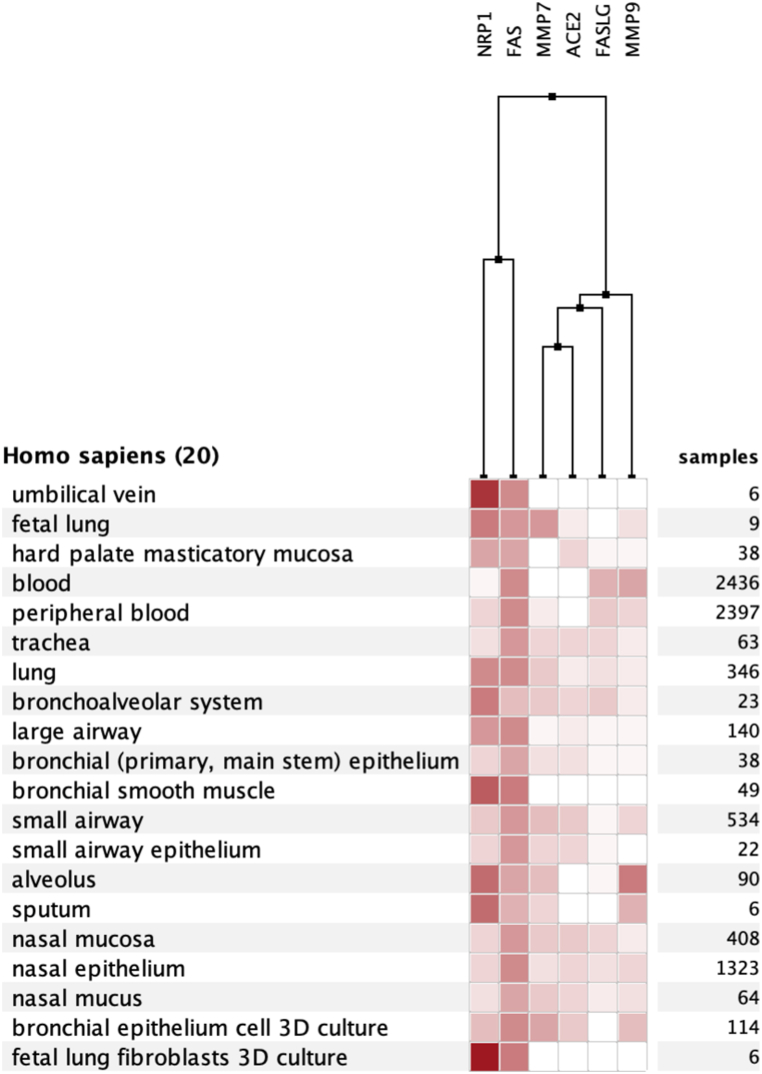
Heatmap expression of MMPs, FasL, and related molecules in specific lung cell types.

**Fig. 10:**
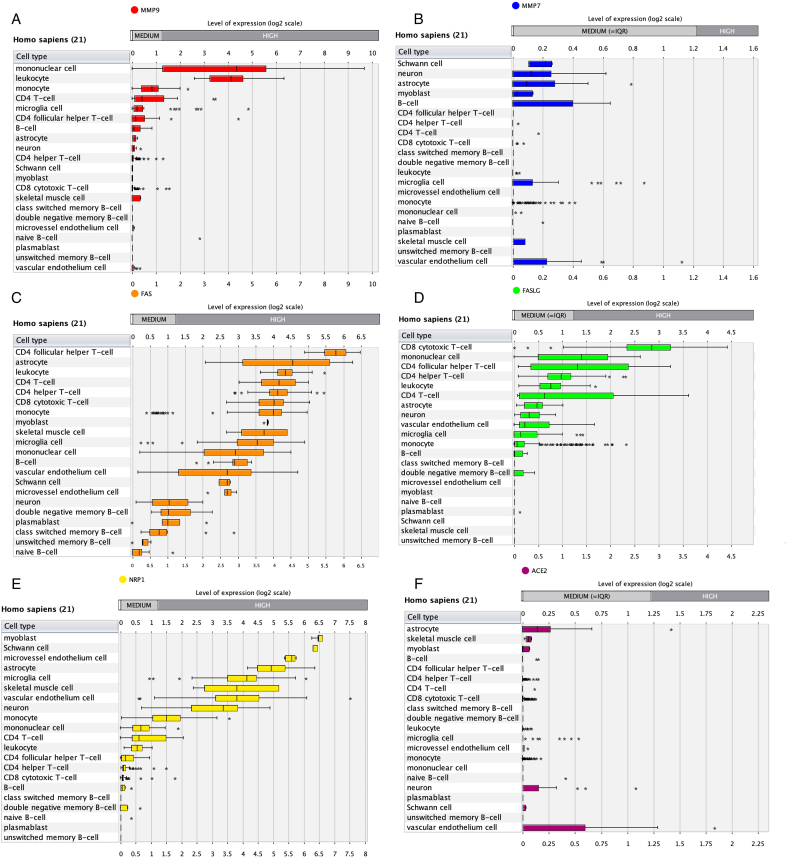
**A-F.** Expression of MMP7/9, FasL, and SARS-CoV2 entry neuroimmune molecules.

## 4 Discussion

MMP7, also called matrilysin, is a protease secreted by various cell types [33]. It regulates cell migration, tissue repair, and innate immunity [34]. MMP7 activates anti-bacterial peptides and releases TNF from macrophages [35]. In healthy adults, MMP9 is found in small amounts in the lung. Various cells within the lung produce MMP9 when stimulated, but most of the research on MMP9 in the lung focuses on its presence in inflammatory cells [36].

MMP7 and MMP9 are two enzymes that have been implicated in the context of COVID-19 by interacting with important molecules such as FasL, the respiratory illness caused by the SARS-CoV-2 virus. MMP7 may serve as an indicator of the continued presence of lung abnormalities after recovering from COVID-19 [36,37]. Elevated levels of MMP7 and MMP9 have been observed in severe cases of COVID-19. A recent study showed one week after admission, obese-diabetic patients with acute respiratory distress syndrome (ARDS) demonstrated notably elevated levels of MMP7 and MMP9 in their serum compared to non-ARDS patients, indicating increased activation of macrophages [38]. In COVID-19, increased MMP7 and MMP9 activity is thought to contribute to lung damage and inflammation through degrading components of the extracellular matrix in the lungs, leading to tissue injury and impaired lung function [39,40].

In another study showed high levels of MMP9 during hospitalization and at 3 months were associated with adverse CT findings [41]. Both MMP7 and MMP9 are being studied as potential biomarkers of disease severity and predictors of clinical outcomes in COVID-19 [42].

FasL is a protein involved in programmed cell death, or apoptosis which can be cleaved into its soluble form (sFasL) by interaction with MMPs [4]. In the context of COVID-19, the role of FasL has been of interest in understanding the immune response and disease progression as it can act as an inflammatory marker. Saleki et al. investigated the role of Fas/FasL) in lung involvement and mortality. we analyzed COVID-19 patients of varying severity and healthy subjects. we found increased mFasL expression in moderate and severe cases and a correlation between reduced lung injury and higher levels of sFasL protein in the serum. Bioinformatics analysis supported these findings. This study suggests that sFasL could be a prognostic marker for COVID-19 severity and mortality, warranting further investigation and potential therapeutic targeting [4]. We discussed a hypothesis that dysregulation of the FasL pathway by cleavage through MMPs may contribute to immune dysregulation, cytokine storm, apoptosis of immune cells, and tissue damage in severe COVID-19 cases [3,43,44].

Neurological sequelae in COVID-19 have been noticed since early 2020 and explored intensely, without a proper molecular explanation [45]. MMP/FasL interactions could be a piece of the neuroimmune interactions leading to cytokine dysregulation in COVID-19. Neuropilin-1 (NRP-1) has been recently proposed as a neuroimmune regulator and viral entry pathway for SARS-CoV2, in addition to the well-known viral entry factor angiotensin-converting enzyme (ACE) 2 [3]. NRP-1 modulates the membrane expression of FasL and MMPs. Cleavage of mFasL to sFasL by various MMPs could lead to increased apoptosis of immune cells and the respiratory system.

Apoptosis clearance failure may lead to increased damage-associated molecular patterns (DAMPs) and activate inflammation-related molecules, such as cytokines, CC-chemokine ligand 2 (CCLs), chemokine (C-X-C motif) ligand 1 (CXCLs), and inflammasomes. Inflammasomes are major regulators of inflammation by governing caspase-1/4/5 mediated production of IL-1 family cytokines [[46], [47], [48], [49]]. The dysregulated cytokines can affect the blood-brain barrier (BBB) accelerating neuroimmunological damage by the SARS-CoV2.

Another area in which MMPs and NRP could cooperate to accelerate COVID-19 damage is angiogenesis which is implicated along with Hypoxia-Inducible Factor-1α (HIF-1α) in COVID-19 [50]. VEGF actions are augmented via co-receptors, like NRP1/2. On the other hand, the loss of VEGF leads to the disruption of vascular development. Placental growth factor (PlGF) is a cytokine VEGF homolog that activates vessel formation and various types of cells, such as myeloids and stromal malignancies, in addition to activating tumor cells, while their inhibition improves cancer treatment [51]. Collagenases are some MMP proteins linked to angiogenesis, and their diminish culminates in non-reversible break down of the matrix. Type IV collagen plays a role in cell endothelial emigration in the interstitial stromal areas. Further, the tissue suppressors of metalloproteinases (TIMP-1/2/3/4) regulate them, exerting a major role in angiogenesis modulation by the blockade of neovascularization [52]. In adults, angiogenesis is induced solely by hyperinflammatory and hypoxia conditions [53]. In the early proliferative stage, vascular repair must take over to control bleeding through vasoconstriction as well as coagulation [54]. These hyperinflammatory and hypoxia-related mechanisms deserve detailed study in COVID-19. Therefore, the role of FasL and MMPs should not be seen as limited to the respiratory system, and research on diverse neuroimmunological mechanisms of COVID-19 is highly recommended.

While there is currently limited information on the specific interaction between MMP7/MMP9 and FasL in COVID-19, it is possible that they may act synergistically or independently in the immune response and tissue damage observed in severe cases of the disease. Further research is needed to elucidate the potential interplay between MMP7/MMP9 and FasL and their contributions to COVID-19 pathogenesis.

Our study provided insights into the interaction between MMP7/MMP9 and FasL in COVID-19. We obtained the protein structures to investigate this interaction. Subsequently, we employed the Patchdock server for docking of FasL with MMP7 and MMP9. To evaluate the impact of MMP7/MMP9 and FasL interaction on the progression of COVID-19, molecular dynamics simulations of the MMP7-FasL and MMP9-FasL complexes were conducted using GROMACS. These simulations were performed under both healthy conditions and COVID-19-like conditions. A limitation is that fever and Na profiles are best personalized for each patient. However, we selected our average temperature and Na according to a real cohort of COVID-19 patients consisting of over 100 mild, moderate, and severe COVID-19 patients. We analyzed bonds, short-range energies, and free binding energies to draw conclusions on the interaction between MMP7/MMP9 and FasL to gain insights into how these complexes could be influenced COVID-19. We found that MMP9 may interact stronger than MMP7 with FasL. However, both these molecules maintained strong interaction through the MDS. Neurobehavioral and neurological conditions are an important public health issue [55]. COVID-19 has been associated with neuropsychiatric and neurological damage. Research has previously demonstrated the neuroimmune involvement of MMP9 in COVID-19. Tsilioni and Theoharides demonstrated that recombinant SARS-CoV2 full-length Spike activates the secretion of pro-inflammatory MMP9 IL-1β/6, and CXCL8 and from cultured human microglia through TLR4 receptor induction. On the other hand, the recombinant receptor-binding domain (RBD) activates the secretion of TNF-α, IL-18, and S100B through ACE2 signaling. Such outcomes provide evidence that SARS-CoV2 spike protein is involved in CNS inflammation by various processes which could be involved in CNS neuropathologies linked to long-COVID [47,56]. Also, Saleki e*t al.* recently performed STRING protein-protein interactions analyses of NRP1, a key entry factor of COVID-19, and showed regulatory pathways and related proteins including inflammatory factors and MMPs. While the MMPs were not the immediate connection of NRP1/VEGF, the analyses showed that associated proteins in the network could include MMPs [57]. Furthermore, as described above in the hypothesis by Saleki et al., cleavage of mFas/mFasL to their soluble forms by MMPs could lead to accelerated cellular apoptosis in the lung, and apoptosis clearance failure could speculatively activate cytokine storm [3]. In line with this hypothesis, research has suggested FasL as an inflammatory compound as well as a mortality and severity prognostic factor in COVID-19. Attenuated sFasL in COVID‐19 cases and as a result decreased signaling to Fas directly enhances RIPK1 levels, worsening TNF‐governed necroptosis. sFasL was also associated with COVID-19 severity, which was alleviated in cases administered with glucocorticoids [4,5]. Together, it is speculated that the roles of MMPs and FasL could be related, and their interactions could be a major regulator of the cytokine storm in COVID-19. Additionally, it is well-known that the end product of these interactions, cytokine storms, may disrupt the blood-brain barrier (BBB). The influx of hyperinflammatory cells is related to MMP synthesis. Viral infections may activate the expression of MMP3 in astrocytes and MMP12 in CNS cells and infiltrating cells. The BBB is somewhat non-permeable in optimal neurophysiological conditions. Buzhdygan and colleagues established a 3D microfluidic simulation of the human BBB and demonstrated that the viral S proteins' activation into the model system leads to BBB permeabilization. Furthermore, SARS-CoV-2 S proteins mediate the MMP3, CCL5, CXCL10, ICAM-1, and VCAM-1 gene expression levels, changing the mRNA levels of IL1-β/6, and activating hyperinflammatory feedback in endothelial cells disrupting the BBB permeability [58,59]. MMP9 which is excreted by infiltrating neutrophils, macrophages, and NK cells is involved in reduced BBB integrity through degrading claudin 5 and ZO-1, consequently regulating influx of hyperinflammatory cells into the CNS. On the other hand, CD8^+^ T-lymphocytes penetrate the parenchyma. This variation in trafficking is because of the expression of tissue inhibitor of MMPs (TIMP-1) in CD4^+^ T-lymphocytes unlike CD8^+^ T-lymphocytes [60]. In COVID-19, MMPs could contribute to BBB disruption. Neutrophils regulate the secretion of MMP9 culminating in increased BBB permeabilization and accelerated penetration of virus-exclusive T-lymphocytes into the CNS. Macrophages can generate MMP9 and excrete type I IFN, restricting viral replication. Also, NK cells produce MMP9 and IFN-γ [[61], [62], [63]]. These neuroimmune factors could accelerate neurological sequelae in COVID-19. While there is some evidence to support such hypotheses, rigorous research is required to investigate the precise neuroimmune involvement by COVID-19.

In this study, we suggested epitopes for MMP-FasL complexes as valuable therapeutic targets in COVID-19. These data could be utilized in future immune drug and protein design efforts. Repurposing of currently approved drugs and herbals could be attempted. Natural products and herbals have been applied to a wide range of disorders [[64], [65], [66], [67], [68], [69], [70], [71], [72], [73]], including COVID-19. Their interaction with MMPs and FasL could be studied in the future through screening, ADMET, MDS, and MM-GBSA techniques using more *in silico* replications and various all-atom force fields. Recently, *in silico* trials designing MMP-mediating candidates have been conducted [74,75], For instance, Katari et al. *designed* MMP-inhibiting compounds by e-pharmacophores designed for the various co-crystal constructs of human matrilysin. These data were mapped based on ligand's pharmacophoric characteristics [74]. In the future, these efforts could be extended to COVID-19 research.

An important benefit of this study is that it emphasizes the expression of mRNA and protein levels may not provide functional insight into the interaction of proteins in COVID-19. *In silico* analyses enable us to investigate precise molecular interactions in modified disease conditions. By combining structural information, docking, and molecular dynamics simulations, we provide insights into molecular interactions of MMPs and FasL, two key molecules in COVID-19. Beyond MMP7/9 we recommend research on more MMP molecules that are involved in COVID-19.

Finally, translational study designs incorporating wet lab and Bioinformatics are recommended to extend our results by experimental validation. For the future, in-depth expression studies on MMPs in patients with COVID-19 are advised.

## 5 Conclusions

Research on the interaction between MMP7/MMP9 and FasL in COVID-19 is still relatively limited. However, our study contributes to the existing body of knowledge by providing valuable insights into the structural aspects and dynamics of these interactions. Using computational approaches, we explored molecular interactions and energies between MMPs and FasL in modified temperature and electrolyte profiles. Also, we suggested MMP/FasL epitopes for targeting when designing immune-mediating therapeutics for COVID-19. MMPs can interact and/or cleave many molecules including Fas/FasL, which makes its implications in multi-organ injury very important. There are many MMPs, and this study shows MMP9 could potentially strongly bind FasL compared to another member of MMP family. When targeting MMPs or related molecules, their cell-specific expression within the CNS, respiratory, and immune cells may be a notable factor to consider. The findings of our study not only enhance our understanding of the molecular mechanisms of MMP/FasL COVID-19, but also pave the way for potential therapeutic interventions targeting the MMP7/MMP9 and FasL pathways. Further research and validation are necessary to extend the significance of our work and develop targeted therapeutic interventions.

## Funding

None.

## Availability of data and materials

Data included in article/supp. material/referenced in the article.

## Ethics approval

Not applicable.

## Consent to participate

Not applicable.

## CRediT authorship contribution statement

**Kiarash Saleki:** Writing – review & editing, Writing – original draft, Visualization, Validation, Supervision, Software, Project administration, Methodology, Investigation, Formal analysis, Data curation, Conceptualization. **Cena Aram:** Writing – original draft, Software, Formal analysis. **Parsa Alijanizadeh:** Writing – review & editing, Writing – original draft, Validation, Software, Data curation. **Mohammad Hossein Khanmirzaei:** Writing – original draft, Visualization, Investigation, Formal analysis, Data curation, Conceptualization. **Zahra Vaziri:** Writing – review & editing, Writing – original draft, Supervision, Software, Formal analysis, Conceptualization. **Mohammad Ramzankhah:** Writing – review & editing, Writing – original draft, Methodology, Conceptualization. **Abbas Azadmehr:** Writing – review & editing, Writing – original draft, Visualization, Project administration, Methodology, Investigation, Formal analysis, Data curation, Conceptualization.

## Declaration of competing interest

The authors declare that they have no known competing financial interests or personal relationships that could have appeared to influence the work reported in this paper.

## Figures and Tables

**Fig. 1a:**
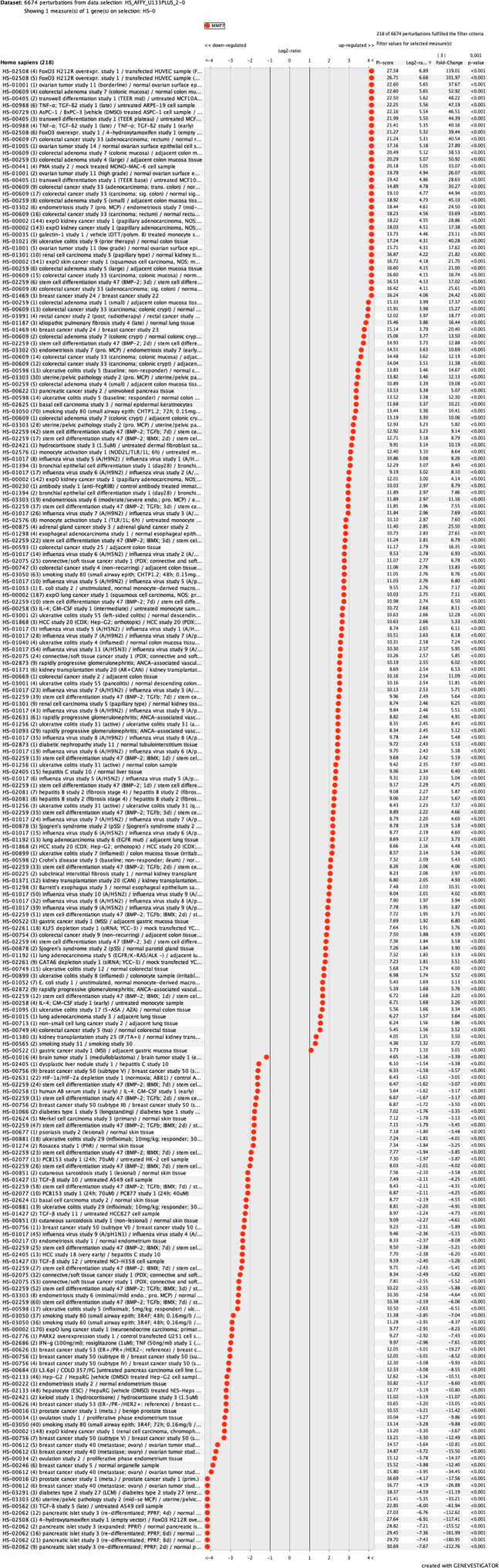
Supp. Condition-specific expression of MMP7.

**Fig. 1b:**
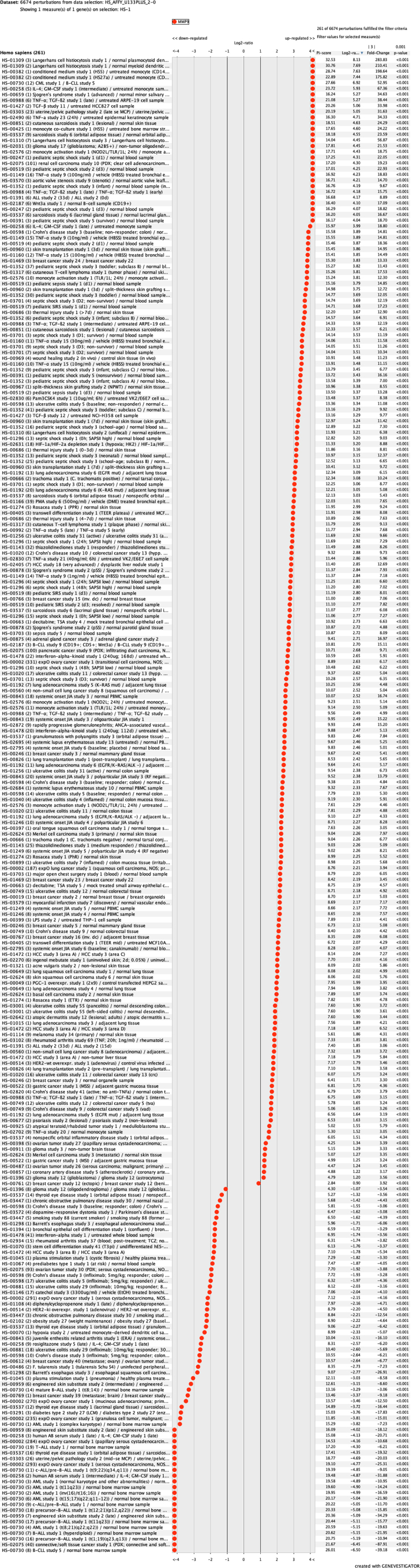
Supp. Condition-specific expression of MMP9.

**Fig. 2a:**
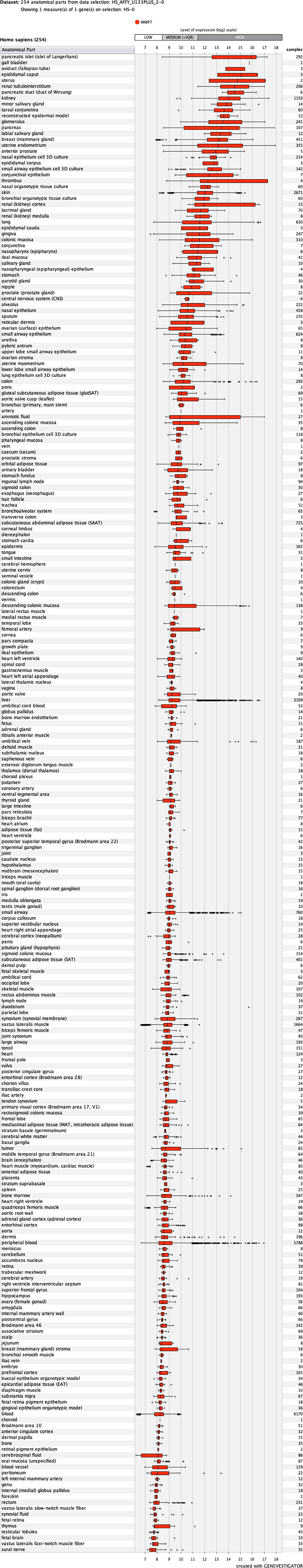
Supp. Tissue-specific expression of MMP7.

**Fig. 2b:**
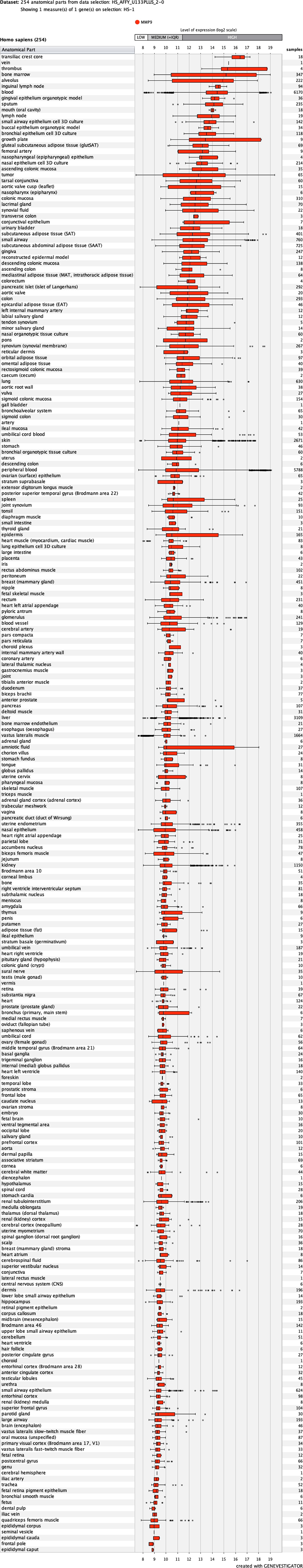
Supp. Tissue-specific expression of MMP9.

**figs5:**
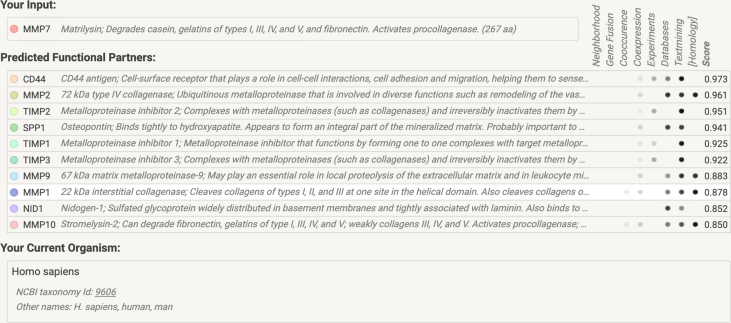


**figs6:**
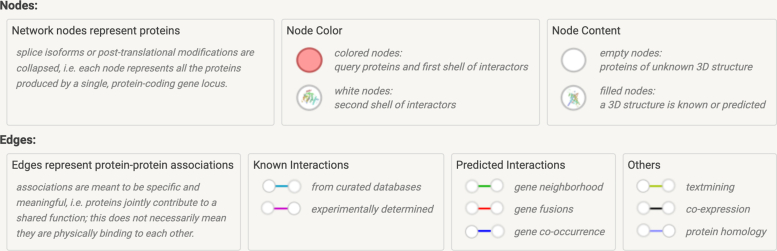


**figs7:**
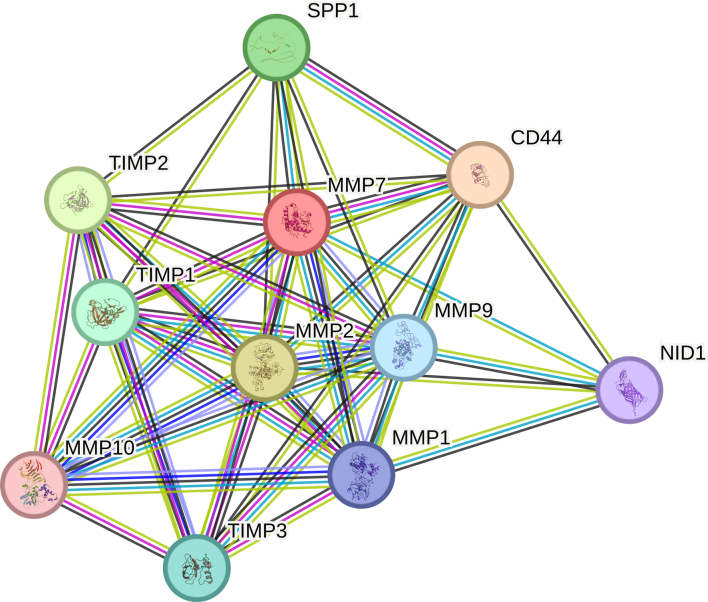


**figs8:**
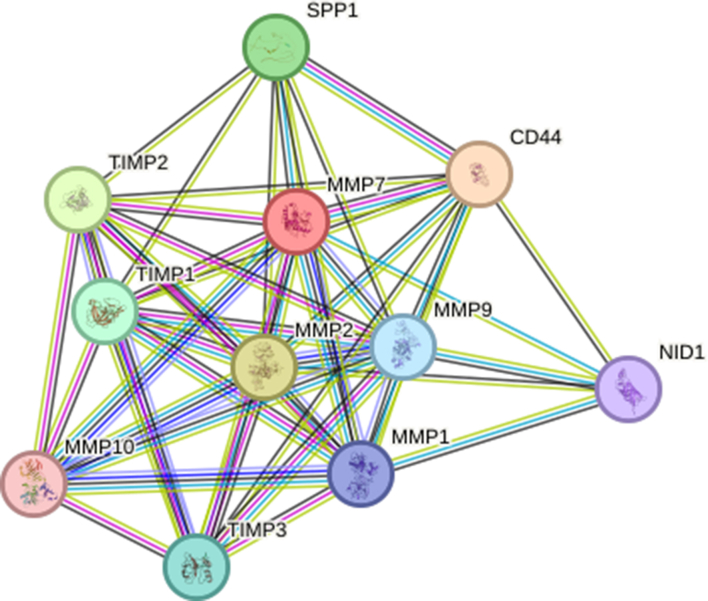


**figs9:**