# Supplementary material for: Matrix metalloproteinase/Fas ligand (MMP/FasL) interaction dynamics in COVID-19: An in silico study and neuroimmune perspective
Source: Heliyon. 2024 May 15;10(10):e30898. doi: 10.1016/j.heliyon.2024.e30898 (PMC11128882; doi:10.1016/j.heliyon.2024.e30898)

**I. Ramachandran Analysis of FasL-MMP7/9 in normal and COVID-19 conditions**

*FasL-MMP7 – normal*

List of atom-atom interactions across protein-protein interface

---------------------------------------------------------------

PDB code: joz4 Chains A }{ B

------------------------------

Hydrogen bonds

--------------

<----- A T O M 1 -----> <----- A T O M 2 ----->

Atom Atom Res Res Atom Atom Res Res

no. name name no. Chain no. name name no. Chain Distance

1. 43 O GLU 3 A <--> 3958 OG1 THR 92 B 2.77

2. 772 O GLN 51 A <--> 3921 N ALA 89 B 3.02

3. 768 NE2 GLN 51 A <--> 4443 ND1 HIS 126 B 2.74

4. 848 OH TYR 55 A <--> 2646 ND2 ASN 8 B 3.15

5. 1134 O ALA 73 A <--> 3810 N GLY 81 B 3.32

6. 1151 NH1 ARG 74 A <--> 3873 O ALA 85 B 2.71

7. 1151 NH1 ARG 74 A <--> 4403 OE1 GLN 123 B 3.13

8. 1159 N ALA 75 A <--> 3830 O ASN 82 B 3.23

9. 1212 OG1 THR 79 A <--> 3739 OH TYR 75 B 2.81

10. 1212 OG1 THR 79 A <--> 3739 OH TYR 75 B 2.81

11. 1294 O CYS 84 A <--> 4684 N TYR 144 B 2.89

12. 1292 SG CYS 84 A <--> 4676 OG1 THR 143 B 3.34

Non-bonded contacts

-------------------

<----- A T O M 1 -----> <----- A T O M 2 ----->

Atom Atom Res Res Atom Atom Res Res

no. name name no. Chain no. name name no. Chain Distance

1. 23 CB ALA 2 A <--> 3986 CD2 LEU 94 B 3.80

2. 43 O GLU 3 A <--> 3956 CB THR 92 B 3.35

3. 43 O GLU 3 A <--> 3958 OG1 THR 92 B 2.77

4. 43 O GLU 3 A <--> 3960 CG2 THR 92 B 3.56

5. 57 O THR 4 A <--> 3960 CG2 THR 92 B 3.84

6. 365 CB PRO 25 A <--> 3915 CE2 PHE 88 B 3.79

7. 738 OH TYR 49 A <--> 4521 ND1 HIS 132 B 2.98

8. 738 OH TYR 49 A <--> 4524 CE1 HIS 132 B 3.25

9. 772 O GLN 51 A <--> 3903 CA PHE 88 B 3.13

10. 772 O GLN 51 A <--> 3919 C PHE 88 B 3.54

11. 772 O GLN 51 A <--> 3905 CB PHE 88 B 3.48

12. 772 O GLN 51 A <--> 3911 CD2 PHE 88 B 3.83

13. 772 O GLN 51 A <--> 3921 N ALA 89 B 3.02

14. 760 CB GLN 51 A <--> 3900 O ALA 87 B 3.83

15. 763 CG GLN 51 A <--> 3900 O ALA 87 B 3.81

16. 766 CD GLN 51 A <--> 3900 O ALA 87 B 3.81

17. 766 CD GLN 51 A <--> 4443 ND1 HIS 126 B 3.80

18. 767 OE1 GLN 51 A <--> 4524 CE1 HIS 132 B 3.82

19. 768 NE2 GLN 51 A <--> 4439 CB HIS 126 B 3.31

20. 768 NE2 GLN 51 A <--> 4442 CG HIS 126 B 3.12

21. 768 NE2 GLN 51 A <--> 4443 ND1 HIS 126 B 2.74

22. 768 NE2 GLN 51 A <--> 4446 CE1 HIS 126 B 3.56

23. 768 NE2 GLN 51 A <--> 4505 O MET 130 B 3.88

24. 783 CD2 PHE 52 A <--> 2668 CG PRO 10 B 3.63

25. 787 CE2 PHE 52 A <--> 2619 CZ PHE 6 B 3.76

26. 789 CZ PHE 52 A <--> 2617 CE2 PHE 6 B 3.70

27. 789 CZ PHE 52 A <--> 2619 CZ PHE 6 B 3.34

28. 848 OH TYR 55 A <--> 2646 ND2 ASN 8 B 3.15

29. 939 NH2 ARG 60 A <--> 2613 CD2 PHE 6 B 3.54

30. 995 CG1 VAL 64 A <--> 4676 OG1 THR 143 B 3.67

31. 999 CG2 VAL 64 A <--> 4659 CB PRO 142 B 3.80

32. 999 CG2 VAL 64 A <--> 4662 CG PRO 142 B 3.84

33. 1023 O LEU 65 A <--> 4676 OG1 THR 143 B 3.25

34. 1124 O GLU 72 A <--> 3810 N GLY 81 B 3.52

35. 1124 O GLU 72 A <--> 3812 CA GLY 81 B 3.58

36. 1133 C ALA 73 A <--> 3810 N GLY 81 B 3.77

37. 1133 C ALA 73 A <--> 3854 CD1 LEU 84 B 3.83

38. 1134 O ALA 73 A <--> 3797 CA PRO 80 B 3.67

39. 1134 O ALA 73 A <--> 3810 N GLY 81 B 3.32

40. 1134 O ALA 73 A <--> 3831 N THR 83 B 3.87

41. 1134 O ALA 73 A <--> 3833 CA THR 83 B 3.79

42. 1134 O ALA 73 A <--> 3843 C THR 83 B 3.88

43. 1134 O ALA 73 A <--> 3854 CD1 LEU 84 B 3.48

44. 1137 CA ARG 74 A <--> 3830 O ASN 82 B 3.49

45. 1157 C ARG 74 A <--> 3830 O ASN 82 B 3.85

46. 1142 CG ARG 74 A <--> 3830 O ASN 82 B 3.69

47. 1142 CG ARG 74 A <--> 3833 CA THR 83 B 3.83

48. 1145 CD ARG 74 A <--> 3837 OG1 THR 83 B 3.64

49. 1145 CD ARG 74 A <--> 3873 O ALA 85 B 3.45

50. 1150 CZ ARG 74 A <--> 3873 O ALA 85 B 3.74

51. 1151 NH1 ARG 74 A <--> 3872 C ALA 85 B 3.75

52. 1151 NH1 ARG 74 A <--> 3873 O ALA 85 B 2.71

53. 1151 NH1 ARG 74 A <--> 3876 CA HIS 86 B 3.70

54. 1151 NH1 ARG 74 A <--> 3878 CB HIS 86 B 3.85

55. 1151 NH1 ARG 74 A <--> 4403 OE1 GLN 123 B 3.13

56. 1159 N ALA 75 A <--> 3830 O ASN 82 B 3.23

57. 1163 CB ALA 75 A <--> 3816 O GLY 81 B 3.88

58. 1188 CD2 HIS 77 A <--> 3730 CD1 TYR 75 B 3.87

59. 1212 OG1 THR 79 A <--> 3734 CE1 TYR 75 B 3.20

60. 1212 OG1 THR 79 A <--> 3738 CZ TYR 75 B 3.40

61. 1212 OG1 THR 79 A <--> 3739 OH TYR 75 B 2.81

62. 1228 ND1 HIS 80 A <--> 3878 CB HIS 86 B 3.27

63. 1228 ND1 HIS 80 A <--> 3881 CG HIS 86 B 3.46

64. 1228 ND1 HIS 80 A <--> 3882 ND1 HIS 86 B 3.44

65. 1231 CE1 HIS 80 A <--> 3734 CE1 TYR 75 B 3.82

66. 1231 CE1 HIS 80 A <--> 3882 ND1 HIS 86 B 3.45

67. 1241 CB ASN 81 A <--> 4524 CE1 HIS 132 B 3.59

68. 1241 CB ASN 81 A <--> 4526 NE2 HIS 132 B 3.88

69. 1246 ND2 ASN 81 A <--> 4520 CG HIS 132 B 3.49

70. 1246 ND2 ASN 81 A <--> 4521 ND1 HIS 132 B 3.57

71. 1246 ND2 ASN 81 A <--> 4522 CD2 HIS 132 B 3.55

72. 1246 ND2 ASN 81 A <--> 4524 CE1 HIS 132 B 3.50

73. 1246 ND2 ASN 81 A <--> 4526 NE2 HIS 132 B 3.75

74. 1274 O ARG 82 A <--> 4659 CB PRO 142 B 3.55

75. 1277 CA ALA 83 A <--> 4669 O PRO 142 B 3.74

76. 1279 CB ALA 83 A <--> 4669 O PRO 142 B 3.65

77. 1294 O CYS 84 A <--> 4672 CA THR 143 B 3.58

78. 1294 O CYS 84 A <--> 4682 C THR 143 B 3.58

79. 1294 O CYS 84 A <--> 4678 CG2 THR 143 B 3.57

80. 1294 O CYS 84 A <--> 4684 N TYR 144 B 2.89

81. 1294 O CYS 84 A <--> 4686 CA TYR 144 B 3.77

82. 1294 O CYS 84 A <--> 4688 CB TYR 144 B 3.38

83. 1292 SG CYS 84 A <--> 4676 OG1 THR 143 B 3.34

84. 1292 SG CYS 84 A <--> 4678 CG2 THR 143 B 3.89

85. 1310 CZ ARG 85 A <--> 3799 CB PRO 80 B 3.80

86. 1310 CZ ARG 85 A <--> 3802 CG PRO 80 B 3.90

87. 1311 NH1 ARG 85 A <--> 3799 CB PRO 80 B 3.86

88. 1311 NH1 ARG 85 A <--> 3802 CG PRO 80 B 3.47

89. 1314 NH2 ARG 85 A <--> 3799 CB PRO 80 B 3.71

90. 1418 CB ALA 92 A <--> 4707 CA GLY 145 B 3.71

91. 1418 CB ALA 92 A <--> 4710 C GLY 145 B 3.37

92. 1418 CB ALA 92 A <--> 4711 O GLY 145 B 3.46

93. 1418 CB ALA 92 A <--> 4712 N ASN 146 B 3.77

94. 1451 N GLY 95 A <--> 4567 CB PRO 136 B 3.68

95. 1451 N GLY 95 A <--> 4570 CG PRO 136 B 3.66

96. 1453 CA GLY 95 A <--> 4567 CB PRO 136 B 3.63

97. 1453 CA GLY 95 A <--> 4570 CG PRO 136 B 3.42

98. 1680 O PRO 112 A <--> 4231 CD1 LEU 112 B 3.14

99. 1694 OG1 THR 114 A <--> 4241 N GLY 113 B 3.52

100. 1694 OG1 THR 114 A <--> 4243 CA GLY 113 B 3.88

101. 1696 CG2 THR 114 A <--> 4224 CA LEU 112 B 3.84

102. 1696 CG2 THR 114 A <--> 4235 CD2 LEU 112 B 3.80

103. 1720 CB SER 116 A <--> 4724 C ASN 146 B 3.79

104. 1720 CB SER 116 A <--> 4725 O ASN 146 B 3.76

105. 1720 CB SER 116 A <--> 4726 N GLY 147 B 3.66

106. 1720 CB SER 116 A <--> 4728 CA GLY 147 B 3.73

107. 1720 CB SER 116 A <--> 4731 C GLY 147 B 3.64

108. 1720 CB SER 116 A <--> 4733 N ASP 148 B 3.30

109. 1723 OG SER 116 A <--> 4728 CA GLY 147 B 3.72

110. 1723 OG SER 116 A <--> 4731 C GLY 147 B 3.37

111. 1723 OG SER 116 A <--> 4732 O GLY 147 B 3.75

112. 1723 OG SER 116 A <--> 4733 N ASP 148 B 3.39

Number of hydrogen bonds: 12

Number of non-bonded contacts: 112

**1. Ramachandran Plot statistics**

**No. of**

**residues %-tage**

**------ ------**

Most favoured regions [A,B,L] 237 83.7%*****

Additional allowed regions [a,b,l,p] 44 15.5%

Generously allowed regions [~a,~b,~l,~p] 1 0.4%

Disallowed regions [XX] 1 0.4%*****

---- ------

Non-glycine and non-proline residues 283 100.0%

End-residues (excl. Gly and Pro) 4

Glycine residues 34

Proline residues 28

----

Total number of residues 349

Based on an analysis of **118** structures of resolution of at least **2.0** Angstroms and *R*-factor no greater than **20.0** a good quality model would be expected to have over **90%** in the most favoured regions [A,B,L].

**2. G-Factors**

**Average**

**Parameter Score Score**

**--------- ----- -----**

Dihedral angles:-

Phi-psi distribution -0.76*

Chi1-chi2 distribution -0.55*

Chi1 only -0.19

Chi3 & chi4 0.54

Omega  **-1.15****

-0.68*

=====

Main-chain covalent forces:-

Main-chain bond lengths  **-1.31****

Main-chain bond angles  **-2.24****

**-1.85****

=====

OVERALL AVERAGE  **-1.11****

=====

**G-factors** provide a measure of how **unusual**, or out-of-the-ordinary, a property is.

Values below -0.5* - unusual

Values below **-1.0**** - highly unusual

**Important note:** The main-chain bond-lengths and bond angles are compared with the Engh & Huber (1991) ideal values derived from small-molecule data. Therefore, structures refined using different restraints may show apparently large deviations from normality.

- Red - Oxygen
- Blue - Nitrogen
- Grey - Carbon
- Yellow - Sulphur
- Blue - Positive (H, K, R)
- Red - Negative (D, E)
- Green - Neutral (S, T, N, Q)
- Grey - Aliphatic (A, V, L, I, M)
- Purple - Aromatic (F, Y, W)
- Orange - Pro & Gly (P, G)
- Yellow - Cysteine (C)
- Red - Most highly conserved
- Pink
- Orange
- Yellow
- Green
- Grey
- Cyan
- Skyblue
- Purple
- Blue - Least conserved

**Aliases for commands entered via console**

+---------------------+

| Binding surfaces |

+---------------------+

protall: shown as wireframe

ligall: shown as spacefill

gap1: Volume = 4560.47 A^3 (red)

gap2: Volume = 2053.69 A^3 (purple)

gap3: Volume = 1475.30 A^3 (yellow)

gap4: Volume = 1255.08 A^3 (blue)

gap5: Volume = 1108.27 A^3 (green)

gap6: Volume = 722.25 A^3 (brown)

gap7: Volume = 648.00 A^3 (pink)

gap8: Volume = 578.39 A^3 (olivegreen)

gap9: Volume = 649.27 A^3 (magenta)

gap10: Volume = 814.64 A^3 (cyan)


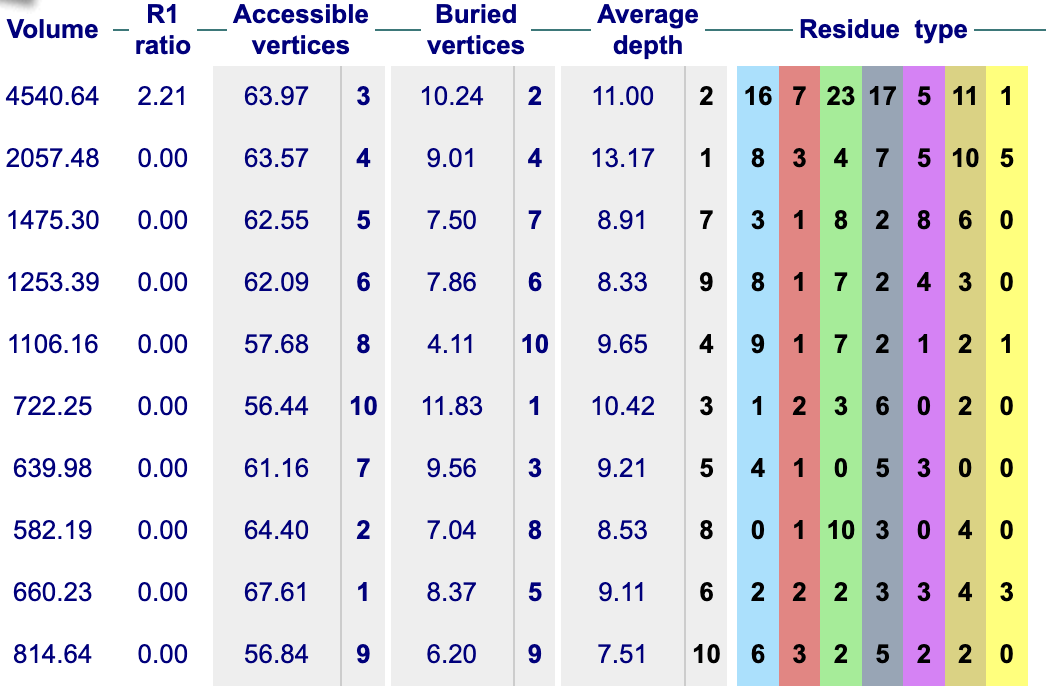


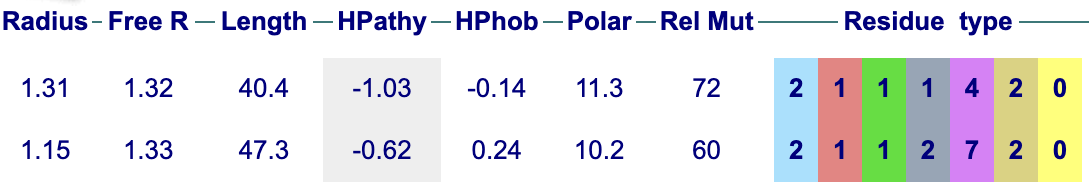


*FasL-MMP7 – COVID-19*

| \| List of atom-atom interactions across protein-protein interface  ---------------------------------------------------------------  PDB code: jpe7 Chains A }{ B  ------------------------------  Hydrogen bonds  --------------  <----- A T O M 1 -----> <----- A T O M 2 ----->  Atom Atom Res Res Atom Atom Res Res  no. name name no. Chain no. name name no. Chain Distance  1. 72 N THR 6 A <--> 3958 OG1 THR 92 B 3.09  2. 1151 NH1 ARG 74 A <--> 3873 O ALA 85 B 3.22  3. 1187 ND1 HIS 77 A <--> 3739 OH TYR 75 B 2.88  4. 1311 NH1 ARG 85 A <--> 4116 OE2 GLU 104 B 2.84  5. 1314 NH2 ARG 85 A <--> 4115 OE1 GLU 104 B 2.71  6. 1440 O HIS 93 A <--> 4726 N GLY 147 B 2.71  7. 1440 O HIS 93 A <--> 4733 N ASP 148 B 3.19  Non-bonded contacts  -------------------  <----- A T O M 1 -----> <----- A T O M 2 ----->  Atom Atom Res Res Atom Atom Res Res  no. name name no. Chain no. name name no. Chain Distance  1. 36 CG GLU 3 A <--> 3965 O THR 92 B 3.43  2. 56 C THR 4 A <--> 3965 O THR 92 B 3.90  3. 57 O THR 4 A <--> 3965 O THR 92 B 3.48  4. 59 CA PRO 5 A <--> 3964 C THR 92 B 3.85  5. 59 CA PRO 5 A <--> 3965 O THR 92 B 3.90  6. 59 CA PRO 5 A <--> 3958 OG1 THR 92 B 3.38  7. 70 C PRO 5 A <--> 3958 OG1 THR 92 B 3.83  8. 61 CB PRO 5 A <--> 3958 OG1 THR 92 B 3.61  9. 72 N THR 6 A <--> 3958 OG1 THR 92 B 3.09  10. 85 O THR 6 A <--> 2646 ND2 ASN 8 B 3.80  11. 80 CG2 THR 6 A <--> 3958 OG1 THR 92 B 3.60  12. 90 CB TYR 7 A <--> 2645 OD1 ASN 8 B 3.50  13. 333 NE2 GLN 22 A <--> 2641 CB ASN 8 B 3.37  14. 333 NE2 GLN 22 A <--> 2644 CG ASN 8 B 3.75  15. 368 CG PRO 25 A <--> 3915 CE2 PHE 88 B 3.89  16. 368 CG PRO 25 A <--> 3917 CZ PHE 88 B 3.74  17. 738 OH TYR 49 A <--> 4521 ND1 HIS 132 B 3.31  18. 738 OH TYR 49 A <--> 4524 CE1 HIS 132 B 3.20  19. 771 C GLN 51 A <--> 3915 CE2 PHE 88 B 3.64  20. 772 O GLN 51 A <--> 2617 CE2 PHE 6 B 3.68  21. 772 O GLN 51 A <--> 3911 CD2 PHE 88 B 3.73  22. 772 O GLN 51 A <--> 3915 CE2 PHE 88 B 3.22  23. 768 NE2 GLN 51 A <--> 3882 ND1 HIS 86 B 3.52  24. 777 CB PHE 52 A <--> 2613 CD2 PHE 6 B 3.63  25. 780 CG PHE 52 A <--> 2632 CD PRO 7 B 3.67  26. 783 CD2 PHE 52 A <--> 2632 CD PRO 7 B 3.44  27. 787 CE2 PHE 52 A <--> 2632 CD PRO 7 B 3.55  28. 816 O TRP 53 A <--> 2626 CB PRO 7 B 3.56  29. 801 CD1 TRP 53 A <--> 3913 CE1 PHE 88 B 3.77  30. 801 CD1 TRP 53 A <--> 3917 CZ PHE 88 B 3.67  31. 843 CE1 TYR 55 A <--> 2626 CB PRO 7 B 3.51  32. 843 CE1 TYR 55 A <--> 2637 N ASN 8 B 3.50  33. 847 CZ TYR 55 A <--> 2637 N ASN 8 B 3.89  34. 848 OH TYR 55 A <--> 2637 N ASN 8 B 3.39  35. 982 CG ASN 63 A <--> 4662 CG PRO 142 B 3.80  36. 983 OD1 ASN 63 A <--> 4662 CG PRO 142 B 3.36  37. 984 ND2 ASN 63 A <--> 4649 CE2 TYR 141 B 3.38  38. 984 ND2 ASN 63 A <--> 4651 CZ TYR 141 B 3.40  39. 984 ND2 ASN 63 A <--> 4652 OH TYR 141 B 3.45  40. 984 ND2 ASN 63 A <--> 4662 CG PRO 142 B 3.44  41. 984 ND2 ASN 63 A <--> 4665 CD PRO 142 B 3.57  42. 999 CG2 VAL 64 A <--> 4669 O PRO 142 B 3.75  43. 1124 O GLU 72 A <--> 3809 O PRO 80 B 3.64  44. 1124 O GLU 72 A <--> 3812 CA GLY 81 B 3.77  45. 1127 CA ALA 73 A <--> 3812 CA GLY 81 B 3.76  46. 1133 C ALA 73 A <--> 3812 CA GLY 81 B 3.84  47. 1133 C ALA 73 A <--> 3815 C GLY 81 B 3.68  48. 1133 C ALA 73 A <--> 3816 O GLY 81 B 3.64  49. 1134 O ALA 73 A <--> 3812 CA GLY 81 B 3.79  50. 1134 O ALA 73 A <--> 3815 C GLY 81 B 3.63  51. 1134 O ALA 73 A <--> 3831 N THR 83 B 3.58  52. 1134 O ALA 73 A <--> 3833 CA THR 83 B 3.42  53. 1134 O ALA 73 A <--> 3843 C THR 83 B 3.18  54. 1134 O ALA 73 A <--> 3844 O THR 83 B 3.21  55. 1134 O ALA 73 A <--> 3845 N LEU 84 B 3.73  56. 1134 O ALA 73 A <--> 3849 CB LEU 84 B 3.79  57. 1135 N ARG 74 A <--> 3816 O GLY 81 B 3.57  58. 1137 CA ARG 74 A <--> 3816 O GLY 81 B 3.79  59. 1137 CA ARG 74 A <--> 3830 O ASN 82 B 3.60  60. 1139 CB ARG 74 A <--> 3833 CA THR 83 B 3.88  61. 1142 CG ARG 74 A <--> 3830 O ASN 82 B 3.52  62. 1142 CG ARG 74 A <--> 3833 CA THR 83 B 3.89  63. 1142 CG ARG 74 A <--> 3837 OG1 THR 83 B 3.88  64. 1145 CD ARG 74 A <--> 3835 CB THR 83 B 3.88  65. 1145 CD ARG 74 A <--> 3837 OG1 THR 83 B 3.26  66. 1151 NH1 ARG 74 A <--> 3873 O ALA 85 B 3.22  67. 1159 N ALA 75 A <--> 3830 O ASN 82 B 3.81  68. 1183 CB HIS 77 A <--> 3739 OH TYR 75 B 3.51  69. 1186 CG HIS 77 A <--> 3736 CE2 TYR 75 B 3.72  70. 1186 CG HIS 77 A <--> 3738 CZ TYR 75 B 3.89  71. 1186 CG HIS 77 A <--> 3739 OH TYR 75 B 3.25  72. 1187 ND1 HIS 77 A <--> 3738 CZ TYR 75 B 3.86  73. 1187 ND1 HIS 77 A <--> 3739 OH TYR 75 B 2.88  74. 1190 CE1 HIS 77 A <--> 3739 OH TYR 75 B 3.65  75. 1196 N ALA 78 A <--> 3739 OH TYR 75 B 3.85  76. 1206 N THR 79 A <--> 3739 OH TYR 75 B 3.46  77. 1214 CG2 THR 79 A <--> 3734 CE1 TYR 75 B 3.63  78. 1214 CG2 THR 79 A <--> 3738 CZ TYR 75 B 3.87  79. 1214 CG2 THR 79 A <--> 3739 OH TYR 75 B 3.49  80. 1228 ND1 HIS 80 A <--> 3729 CG TYR 75 B 3.85  81. 1228 ND1 HIS 80 A <--> 3730 CD1 TYR 75 B 3.48  82. 1228 ND1 HIS 80 A <--> 3734 CE1 TYR 75 B 3.82  83. 1231 CE1 HIS 80 A <--> 3726 CB TYR 75 B 3.86  84. 1231 CE1 HIS 80 A <--> 3729 CG TYR 75 B 3.38  85. 1231 CE1 HIS 80 A <--> 3730 CD1 TYR 75 B 3.47  86. 1231 CE1 HIS 80 A <--> 3732 CD2 TYR 75 B 3.77  87. 1231 CE1 HIS 80 A <--> 3734 CE1 TYR 75 B 3.77  88. 1231 CE1 HIS 80 A <--> 3830 O ASN 82 B 3.64  89. 1233 NE2 HIS 80 A <--> 3734 CE1 TYR 75 B 3.86  90. 1233 NE2 HIS 80 A <--> 3736 CE2 TYR 75 B 3.88  91. 1233 NE2 HIS 80 A <--> 3738 CZ TYR 75 B 3.87  92. 1279 CB ALA 83 A <--> 3858 CD2 LEU 84 B 3.62  93. 1279 CB ALA 83 A <--> 4692 CD1 TYR 144 B 3.75  94. 1310 CZ ARG 85 A <--> 4114 CD GLU 104 B 3.74  95. 1310 CZ ARG 85 A <--> 4115 OE1 GLU 104 B 3.50  96. 1310 CZ ARG 85 A <--> 4116 OE2 GLU 104 B 3.69  97. 1311 NH1 ARG 85 A <--> 4114 CD GLU 104 B 3.24  98. 1311 NH1 ARG 85 A <--> 4115 OE1 GLU 104 B 3.47  99. 1311 NH1 ARG 85 A <--> 4116 OE2 GLU 104 B 2.84  100. 1311 NH1 ARG 85 A <--> 4254 CG1 ILE 114 B 3.42  101. 1314 NH2 ARG 85 A <--> 4114 CD GLU 104 B 3.37  102. 1314 NH2 ARG 85 A <--> 4115 OE1 GLU 104 B 2.71  103. 1314 NH2 ARG 85 A <--> 4116 OE2 GLU 104 B 3.59  104. 1418 CB ALA 92 A <--> 4711 O GLY 145 B 3.30  105. 1418 CB ALA 92 A <--> 4726 N GLY 147 B 3.84  106. 1439 C HIS 93 A <--> 4726 N GLY 147 B 3.83  107. 1439 C HIS 93 A <--> 4733 N ASP 148 B 3.72  108. 1440 O HIS 93 A <--> 4714 CA ASN 146 B 3.51  109. 1440 O HIS 93 A <--> 4724 C ASN 146 B 3.57  110. 1440 O HIS 93 A <--> 4719 CG ASN 146 B 3.65  111. 1440 O HIS 93 A <--> 4720 OD1 ASN 146 B 3.26  112. 1440 O HIS 93 A <--> 4726 N GLY 147 B 2.71  113. 1440 O HIS 93 A <--> 4728 CA GLY 147 B 3.59  114. 1440 O HIS 93 A <--> 4731 C GLY 147 B 3.70  115. 1440 O HIS 93 A <--> 4733 N ASP 148 B 3.19  116. 1443 CA ALA 94 A <--> 4719 CG ASN 146 B 3.89  117. 1443 CA ALA 94 A <--> 4720 OD1 ASN 146 B 3.71  118. 1443 CA ALA 94 A <--> 4721 ND2 ASN 146 B 3.80  119. 1449 C ALA 94 A <--> 4721 ND2 ASN 146 B 3.73  120. 1451 N GLY 95 A <--> 4719 CG ASN 146 B 3.85  121. 1451 N GLY 95 A <--> 4721 ND2 ASN 146 B 3.80  122. 1453 CA GLY 95 A <--> 4711 O GLY 145 B 3.63  123. 1456 C GLY 95 A <--> 4711 O GLY 145 B 3.50  124. 1457 O GLY 95 A <--> 4711 O GLY 145 B 3.42  125. 1708 CG PRO 115 A <--> 4247 O GLY 113 B 3.82  126. 1711 CD PRO 115 A <--> 4221 O SER 111 B 3.58  127. 1726 O SER 116 A <--> 4732 O GLY 147 B 3.71  128. 1720 CB SER 116 A <--> 4728 CA GLY 147 B 3.67  Salt bridges  ------------  <----- A T O M 1 -----> <----- A T O M 2 ----->  Atom Atom Res Res Atom Atom Res Res  no. name name no. Chain no. name name no. Chain Distance  1. 1314 NH2 ARG 85 A <--> 4115 OE1 GLU 104 B 2.71  Number of salt bridges: 1  Number of hydrogen bonds: 7  Number of non-bonded contacts: 128  **PROCHECK statistics**  **1. Ramachandran Plot statistics**  **No. of**  **residues %-tage**  **------ ------**  Most favoured regions [A,B,L] 226 79.9%******  Additional allowed regions [a,b,l,p] 52 18.4%  Generously allowed regions [~a,~b,~l,~p] 5 1.8%  Disallowed regions [XX] 0 0.0%  ---- ------  Non-glycine and non-proline residues 283 100.0%  End-residues (excl. Gly and Pro) 4  Glycine residues 34  Proline residues 28  ----  Total number of residues 349  Based on an analysis of **118** structures of resolution of at least **2.0** Angstroms and *R*-factor no greater than **20.0** a good quality model would be expected to have over **90%** in the most favoured regions [A,B,L].  **2. G-Factors**  **Average**  **Parameter Score Score**  **--------- ----- -----**  Dihedral angles:-  Phi-psi distribution -0.85*  Chi1-chi2 distribution -0.60*  Chi1 only 0.07  Chi3 & chi4 0.47  Omega  **-1.04****  -0.67*  =====  Main-chain covalent forces:-  Main-chain bond lengths  **-1.26****  Main-chain bond angles  **-2.25****  **-1.83****  =====  OVERALL AVERAGE  **-1.10****  =====  **G-factors** provide a measure of how **unusual**, or out-of-the-ordinary, a property is.  Values below -0.5* - unusual  Values below **-1.0**** - highly unusual  **Important note:** The main-chain bond-lengths and bond angles are compared with the Engh & Huber (1991) ideal values derived from small-molecule data. Therefore, structures refined using different restraints may show apparently large deviations from normality.  **Aliases for commands entered via console**    +---------------------+  \| Binding sites \|  +---------------------+    protall: shown as wireframe  ligall: shown as spacefill  gap1: Volume = 3193.17 A^3 (red)  gap2: Volume = 3269.11 A^3 (purple)  gap3: Volume = 2229.61 A^3 (yellow)  gap4: Volume = 1337.77 A^3 (blue)  gap5: Volume = 1117.12 A^3 (green)  gap6: Volume = 715.08 A^3 (brown)  gap7: Volume = 603.70 A^3 (pink)  gap8: Volume = 877.08 A^3 (olivegreen)  gap9: Volume = 820.97 A^3 (magenta)  gap10: Volume = 551.39 A^3 (cyan) \| \| --- \| |
| --- | --- |


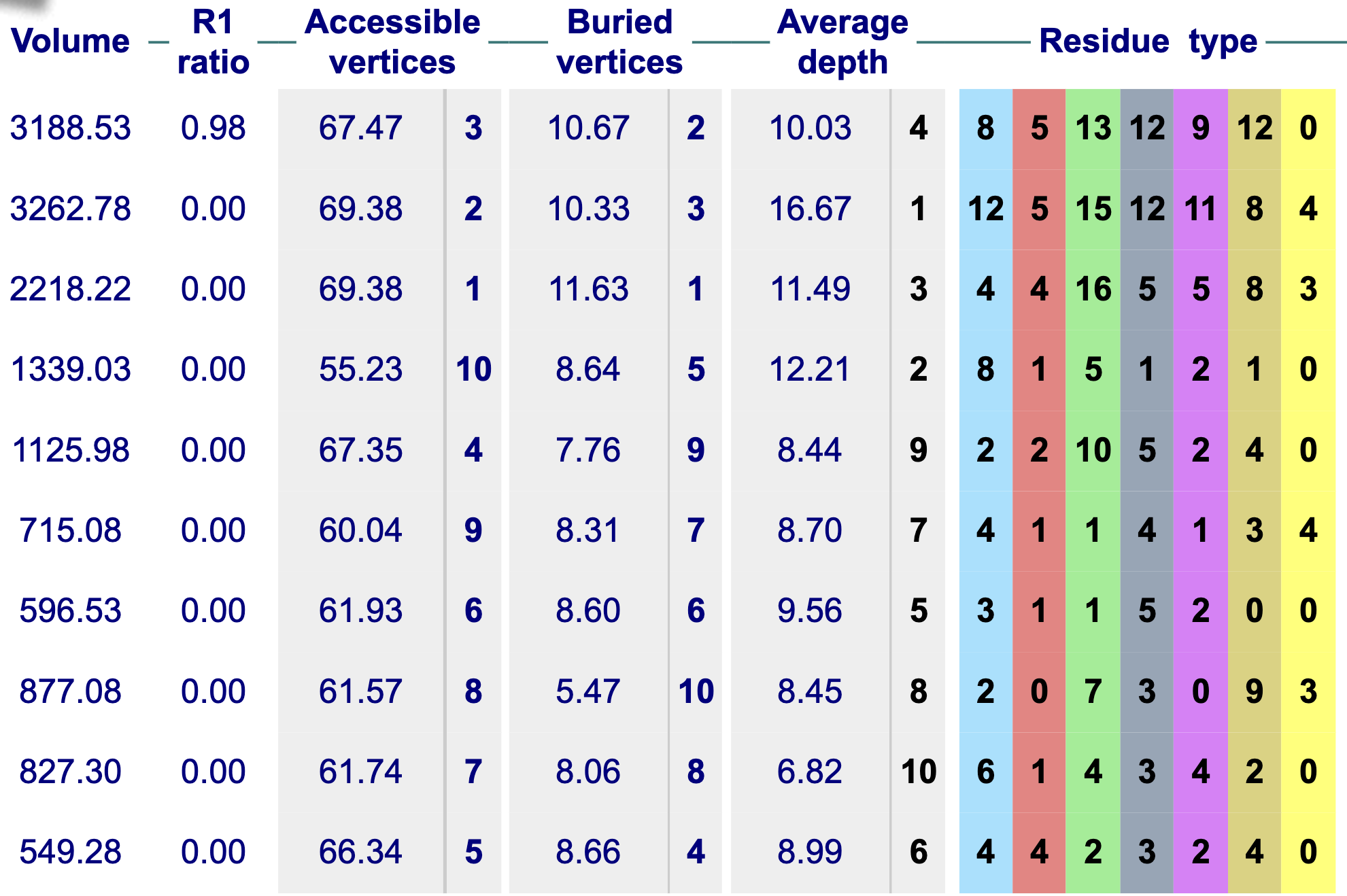


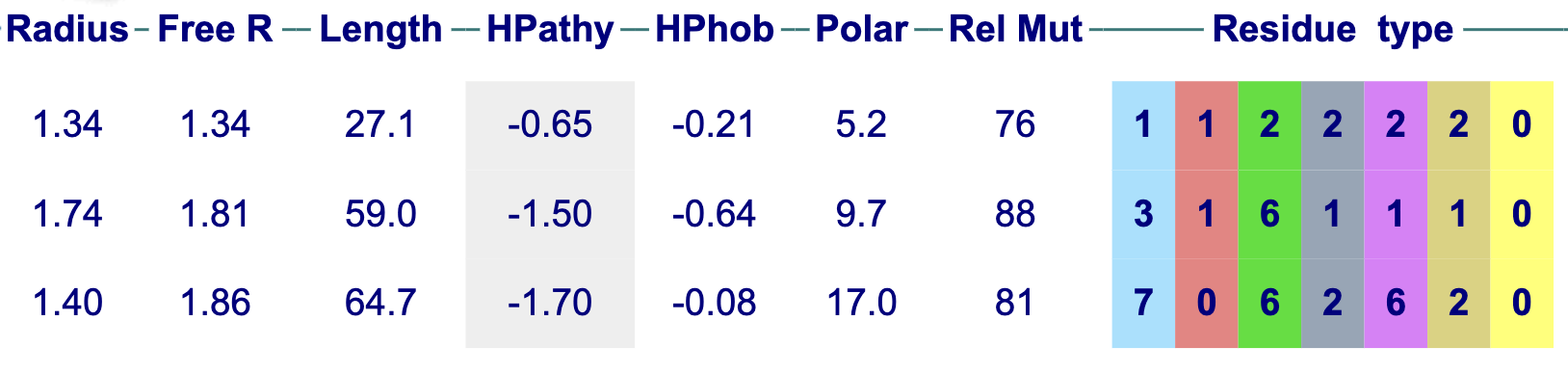


*FasL-MMP9 Healthy*

PDB code: jpf2 Chains A }{ B

------------------------------

Hydrogen bonds

--------------

<----- A T O M 1 -----> <----- A T O M 2 ----->

Atom Atom Res Res Atom Atom Res Res

no. name name no. Chain no. name name no. Chain Distance

1. 161 NH1 ARG 10 A <--> 4001 OE2 GLU 92 B 2.55

2. 164 NH2 ARG 10 A <--> 4000 OE1 GLU 92 B 2.81

3. 738 OH TYR 49 A <--> 8427 OD1 ASP 391 B 2.53

4. 848 OH TYR 55 A <--> 4010 O GLY 93 B 2.80

5. 939 NH2 ARG 60 A <--> 8399 O GLY 389 B 2.80

6. 984 ND2 ASN 63 A <--> 8811 O ASP 415 B 2.84

7. 984 ND2 ASN 63 A <--> 8823 O ASP 416 B 2.91

8. 1308 NE ARG 85 A <--> 3558 OE1 GLU 63 B 2.88

9. 1314 NH2 ARG 85 A <--> 3559 OE2 GLU 63 B 2.79

10. 1811 NE2 GLN 122 A <--> 2575 O ARG 3 B 2.71

11. 1811 NE2 GLN 122 A <--> 2628 OE1 GLN 6 B 2.93

Non-bonded contacts

-------------------

<----- A T O M 1 -----> <----- A T O M 2 ----->

Atom Atom Res Res Atom Atom Res Res

no. name name no. Chain no. name name no. Chain Distance

1. 64 CG PRO 5 A <--> 3913 NH2 ARG 87 B 3.85

2. 85 O THR 6 A <--> 3963 CG2 THR 90 B 3.43

3. 113 CG PRO 8 A <--> 3988 O PHE 91 B 3.71

4. 139 CZ3 TRP 9 A <--> 4010 O GLY 93 B 3.86

5. 160 CZ ARG 10 A <--> 4000 OE1 GLU 92 B 3.35

6. 160 CZ ARG 10 A <--> 4001 OE2 GLU 92 B 3.59

7. 161 NH1 ARG 10 A <--> 3999 CD GLU 92 B 3.18

8. 161 NH1 ARG 10 A <--> 4000 OE1 GLU 92 B 3.06

9. 161 NH1 ARG 10 A <--> 4001 OE2 GLU 92 B 2.55

10. 164 NH2 ARG 10 A <--> 3999 CD GLU 92 B 3.74

11. 164 NH2 ARG 10 A <--> 4000 OE1 GLU 92 B 2.81

12. 164 NH2 ARG 10 A <--> 4001 OE2 GLU 92 B 3.84

13. 261 NH2 ARG 17 A <--> 4000 OE1 GLU 92 B 3.59

14. 333 NE2 GLN 22 A <--> 3963 CG2 THR 90 B 3.79

15. 735 CE2 TYR 49 A <--> 8427 OD1 ASP 391 B 3.79

16. 737 CZ TYR 49 A <--> 8427 OD1 ASP 391 B 3.56

17. 738 OH TYR 49 A <--> 8423 CB ASP 391 B 3.68

18. 738 OH TYR 49 A <--> 8426 CG ASP 391 B 3.49

19. 738 OH TYR 49 A <--> 8427 OD1 ASP 391 B 2.53

20. 767 OE1 GLN 51 A <--> 3875 CG LEU 85 B 3.78

21. 767 OE1 GLN 51 A <--> 3877 CD1 LEU 85 B 3.59

22. 768 NE2 GLN 51 A <--> 8427 OD1 ASP 391 B 3.86

23. 780 CG PHE 52 A <--> 3904 CD ARG 87 B 3.83

24. 783 CD2 PHE 52 A <--> 3904 CD ARG 87 B 3.68

25. 785 CE1 PHE 52 A <--> 3893 O GLY 86 B 3.74

26. 801 CD1 TRP 53 A <--> 3913 NH2 ARG 87 B 3.61

27. 845 CE2 TYR 55 A <--> 4010 O GLY 93 B 3.51

28. 845 CE2 TYR 55 A <--> 4013 CA ASP 94 B 3.76

29. 847 CZ TYR 55 A <--> 4010 O GLY 93 B 3.56

30. 848 OH TYR 55 A <--> 3979 CD2 PHE 91 B 3.69

31. 848 OH TYR 55 A <--> 3983 CE2 PHE 91 B 3.02

32. 848 OH TYR 55 A <--> 3985 CZ PHE 91 B 3.43

33. 848 OH TYR 55 A <--> 4009 C GLY 93 B 3.87

34. 848 OH TYR 55 A <--> 4010 O GLY 93 B 2.80

35. 883 OE2 GLU 57 A <--> 4058 NZ LYS 96 B 3.85

36. 909 O ARG 58 A <--> 8913 CD2 HIS 422 B 3.69

37. 890 CB ARG 58 A <--> 8920 O HIS 422 B 3.29

38. 893 CG ARG 58 A <--> 8920 O HIS 422 B 3.79

39. 896 CD ARG 58 A <--> 8920 O HIS 422 B 3.38

40. 899 NE ARG 58 A <--> 8919 C HIS 422 B 3.90

41. 899 NE ARG 58 A <--> 8920 O HIS 422 B 3.11

42. 901 CZ ARG 58 A <--> 8920 O HIS 422 B 3.51

43. 902 NH1 ARG 58 A <--> 8903 O ARG 421 B 3.78

44. 902 NH1 ARG 58 A <--> 8966 C GLY 425 B 3.68

45. 905 NH2 ARG 58 A <--> 8939 O LEU 423 B 3.38

46. 919 O CYS 59 A <--> 8908 CB HIS 422 B 3.41

47. 919 O CYS 59 A <--> 8911 CG HIS 422 B 3.76

48. 927 CG ARG 60 A <--> 8917 NE2 HIS 422 B 3.74

49. 933 NE ARG 60 A <--> 8917 NE2 HIS 422 B 3.34

50. 935 CZ ARG 60 A <--> 8399 O GLY 389 B 3.75

51. 935 CZ ARG 60 A <--> 8423 CB ASP 391 B 3.83

52. 935 CZ ARG 60 A <--> 8917 NE2 HIS 422 B 3.60

53. 936 NH1 ARG 60 A <--> 4036 CD2 LEU 95 B 3.76

54. 936 NH1 ARG 60 A <--> 8399 O GLY 389 B 3.75

55. 939 NH2 ARG 60 A <--> 8398 C GLY 389 B 3.80

56. 939 NH2 ARG 60 A <--> 8399 O GLY 389 B 2.80

57. 939 NH2 ARG 60 A <--> 8402 CA LEU 390 B 3.84

58. 939 NH2 ARG 60 A <--> 8417 C LEU 390 B 3.78

59. 939 NH2 ARG 60 A <--> 8419 N ASP 391 B 3.41

60. 939 NH2 ARG 60 A <--> 8423 CB ASP 391 B 3.81

61. 939 NH2 ARG 60 A <--> 8915 CE1 HIS 422 B 3.88

62. 939 NH2 ARG 60 A <--> 8917 NE2 HIS 422 B 3.54

63. 954 CD2 TYR 61 A <--> 8848 OD1 ASN 418 B 3.89

64. 958 CE2 TYR 61 A <--> 8848 OD1 ASN 418 B 3.82

65. 988 O ASN 63 A <--> 8811 O ASP 415 B 3.11

66. 982 CG ASN 63 A <--> 8811 O ASP 415 B 3.88

67. 982 CG ASN 63 A <--> 8854 N GLY 419 B 3.82

68. 982 CG ASN 63 A <--> 8856 CA GLY 419 B 3.73

69. 983 OD1 ASN 63 A <--> 8856 CA GLY 419 B 3.63

70. 984 ND2 ASN 63 A <--> 8810 C ASP 415 B 3.80

71. 984 ND2 ASN 63 A <--> 8811 O ASP 415 B 2.84

72. 984 ND2 ASN 63 A <--> 8814 CA ASP 416 B 3.58

73. 984 ND2 ASN 63 A <--> 8822 C ASP 416 B 3.51

74. 984 ND2 ASN 63 A <--> 8823 O ASP 416 B 2.91

75. 984 ND2 ASN 63 A <--> 8854 N GLY 419 B 3.66

76. 995 CG1 VAL 64 A <--> 8480 CG2 VAL 395 B 3.83

77. 1127 CA ALA 73 A <--> 3558 OE1 GLU 63 B 3.68

78. 1134 O ALA 73 A <--> 3455 CD LYS 57 B 3.69

79. 1134 O ALA 73 A <--> 3458 CE LYS 57 B 3.64

80. 1129 CB ALA 73 A <--> 3466 O LYS 57 B 3.89

81. 1151 NH1 ARG 74 A <--> 3403 CD2 LEU 54 B 3.75

82. 1274 O ARG 82 A <--> 8466 OG SER 394 B 3.70

83. 1277 CA ALA 83 A <--> 8466 OG SER 394 B 3.13

84. 1279 CB ALA 83 A <--> 3466 O LYS 57 B 3.83

85. 1279 CB ALA 83 A <--> 8466 OG SER 394 B 3.27

86. 1294 O CYS 84 A <--> 8476 CG1 VAL 395 B 3.62

87. 1292 SG CYS 84 A <--> 8480 CG2 VAL 395 B 3.49

88. 1299 CB ARG 85 A <--> 3512 C SER 60 B 3.86

89. 1299 CB ARG 85 A <--> 3513 O SER 60 B 3.34

90. 1299 CB ARG 85 A <--> 3507 CB SER 60 B 3.82

91. 1305 CD ARG 85 A <--> 3512 C SER 60 B 3.74

92. 1305 CD ARG 85 A <--> 3513 O SER 60 B 3.41

93. 1305 CD ARG 85 A <--> 3531 C LEU 61 B 3.51

94. 1305 CD ARG 85 A <--> 3532 O LEU 61 B 3.02

95. 1305 CD ARG 85 A <--> 3558 OE1 GLU 63 B 3.70

96. 1308 NE ARG 85 A <--> 3531 C LEU 61 B 3.77

97. 1308 NE ARG 85 A <--> 3532 O LEU 61 B 3.25

98. 1308 NE ARG 85 A <--> 3533 N PRO 62 B 3.84

99. 1308 NE ARG 85 A <--> 3557 CD GLU 63 B 3.22

100. 1308 NE ARG 85 A <--> 3558 OE1 GLU 63 B 2.88

101. 1308 NE ARG 85 A <--> 3559 OE2 GLU 63 B 3.35

102. 1310 CZ ARG 85 A <--> 3532 O LEU 61 B 3.88

103. 1310 CZ ARG 85 A <--> 3533 N PRO 62 B 3.73

104. 1310 CZ ARG 85 A <--> 3534 CA PRO 62 B 3.64

105. 1310 CZ ARG 85 A <--> 3557 CD GLU 63 B 3.76

106. 1310 CZ ARG 85 A <--> 3558 OE1 GLU 63 B 3.61

107. 1310 CZ ARG 85 A <--> 3559 OE2 GLU 63 B 3.44

108. 1311 NH1 ARG 85 A <--> 3533 N PRO 62 B 3.67

109. 1311 NH1 ARG 85 A <--> 3534 CA PRO 62 B 3.88

110. 1314 NH2 ARG 85 A <--> 3534 CA PRO 62 B 3.77

111. 1314 NH2 ARG 85 A <--> 3557 CD GLU 63 B 3.48

112. 1314 NH2 ARG 85 A <--> 3558 OE1 GLU 63 B 3.53

113. 1314 NH2 ARG 85 A <--> 3559 OE2 GLU 63 B 2.79

114. 1326 SG CYS 86 A <--> 8492 CG PRO 396 B 3.77

115. 1443 CA ALA 94 A <--> 8686 CG2 THR 407 B 3.85

116. 1449 C ALA 94 A <--> 8686 CG2 THR 407 B 3.67

117. 1450 O ALA 94 A <--> 8686 CG2 THR 407 B 3.61

118. 1451 N GLY 95 A <--> 8650 NH1 ARG 405 B 3.90

119. 1453 CA GLY 95 A <--> 8499 O PRO 396 B 3.74

120. 1453 CA GLY 95 A <--> 8650 NH1 ARG 405 B 3.48

121. 1456 C GLY 95 A <--> 8499 O PRO 396 B 3.87

122. 1457 O GLY 95 A <--> 8502 CA GLU 397 B 3.75

123. 1457 O GLY 95 A <--> 8731 CB PRO 411 B 3.66

124. 1457 O GLY 95 A <--> 8734 CG PRO 411 B 3.62

125. 1705 CB PRO 115 A <--> 3542 CD PRO 62 B 3.57

126. 1708 CG PRO 115 A <--> 3542 CD PRO 62 B 3.51

127. 1708 CG PRO 115 A <--> 3644 CB ALA 70 B 3.84

128. 1711 CD PRO 115 A <--> 3644 CB ALA 70 B 3.85

129. 1784 NE2 GLN 120 A <--> 2544 CG PRO 2 B 3.43

130. 1803 CB GLN 122 A <--> 2575 O ARG 3 B 3.45

131. 1809 CD GLN 122 A <--> 2575 O ARG 3 B 3.79

132. 1809 CD GLN 122 A <--> 2628 OE1 GLN 6 B 3.66

133. 1810 OE1 GLN 122 A <--> 2628 OE1 GLN 6 B 3.58

134. 1811 NE2 GLN 122 A <--> 2574 C ARG 3 B 3.88

135. 1811 NE2 GLN 122 A <--> 2575 O ARG 3 B 2.71

136. 1811 NE2 GLN 122 A <--> 2592 O GLN 4 B 3.89

137. 1811 NE2 GLN 122 A <--> 2627 CD GLN 6 B 3.86

138. 1811 NE2 GLN 122 A <--> 2628 OE1 GLN 6 B 2.93

139. 1829 O PRO 123 A <--> 2578 CA GLN 4 B 3.56

140. 1822 CG PRO 123 A <--> 2576 N GLN 4 B 3.84

141. 1841 CA PRO 125 A <--> 2587 OE1 GLN 4 B 3.69

142. 1863 CD PRO 126 A <--> 2587 OE1 GLN 4 B 3.38

Salt bridges

------------

<----- A T O M 1 -----> <----- A T O M 2 ----->

Atom Atom Res Res Atom Atom Res Res

no. name name no. Chain no. name name no. Chain Distance

1. 161 NH1 ARG 10 A <--> 4001 OE2 GLU 92 B 2.55

2. 261 NH2 ARG 17 A <--> 4000 OE1 GLU 92 B 3.59

3. 883 OE2 GLU 57 A <--> 4058 NZ LYS 96 B 3.85

4. 1314 NH2 ARG 85 A <--> 3559 OE2 GLU 63 B 2.79

Number of salt bridges: 4

Number of hydrogen bonds: 11

Number of non-bonded contacts: 142

## 1. Ramachandran Plot statistics

List of atom-atom interactions across protein-protein interface

---------------------------------------------------------------

**No. of**

**residues %-tage**

**------ ------**

Most favoured regions [A,B,L] 380 76.6%******

Additional allowed regions [a,b,l,p] 108 21.8%

Generously allowed regions [~a,~b,~l,~p] 7 1.4%

Disallowed regions [XX] 1 0.2%*****

---- ------

Non-glycine and non-proline residues 496 100.0%

End-residues (excl. Gly and Pro) 3

Glycine residues 57

Proline residues 43

----

Total number of residues 599

Based on an analysis of **118** structures of resolution of at least **2.0** Angstroms and R-factor no greater than **20.0** a good quality model would be expected to have over **90%** in the most favoured regions [A,B,L].

## 2. G-Factors

**Average**

**Parameter Score Score**

**--------- ----- -----**

Dihedral angles:-

Phi-psi distribution -0.85*

Chi1-chi2 distribution -0.57*

Chi1 only -0.12

Chi3 & chi4 0.48

Omega  **-1.17****

-0.72*

=====

Main-chain covalent forces:-

Main-chain bond lengths  **-1.30****

Main-chain bond angles  **-2.21****

**-1.83****

=====

OVERALL AVERAGE  **-1.13****

=====

**G-factors** provide a measure of how **unusual**, or out-of-the-ordinary, a property is.

Values below -0.5* - unusual

Values below **-1.0**** - highly unusual

**Important note:** The main-chain bond-lengths and bond angles are compared with the Engh & Huber (1991) ideal values derived from small-molecule data. Therefore, structures refined using different restraints may show apparently large deviations from normality.

*A part of interactions was not included due to figure size restriction. Here, full interactions are plotted.


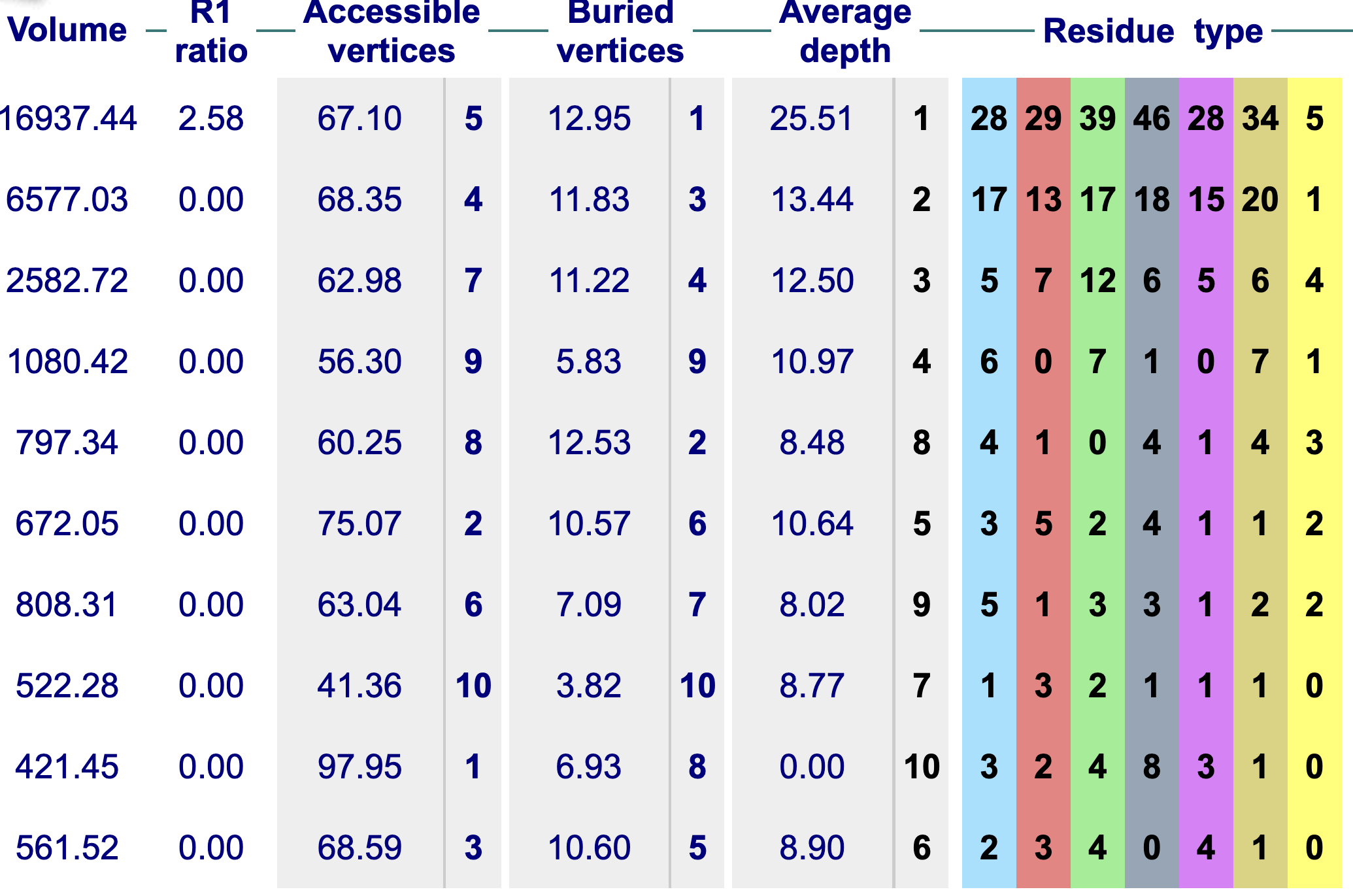


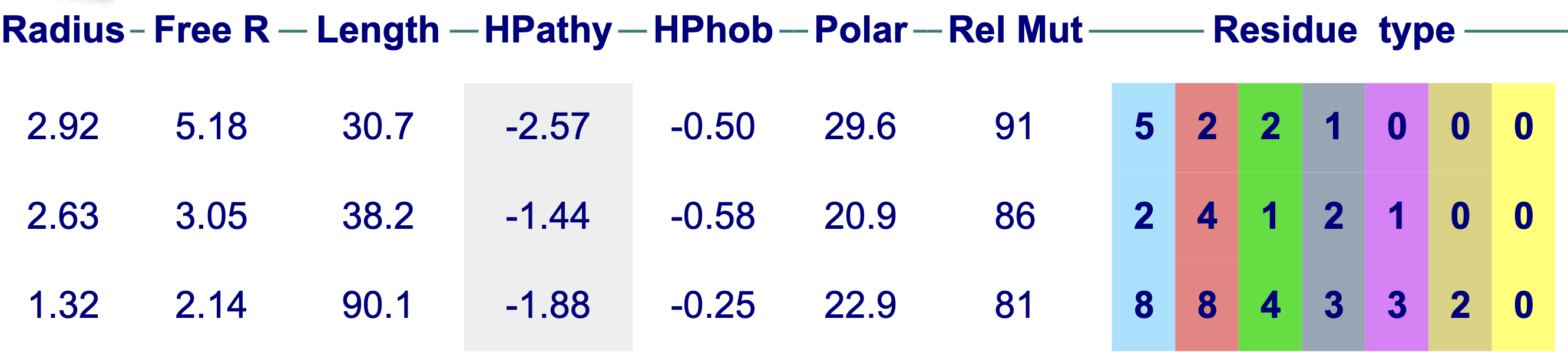


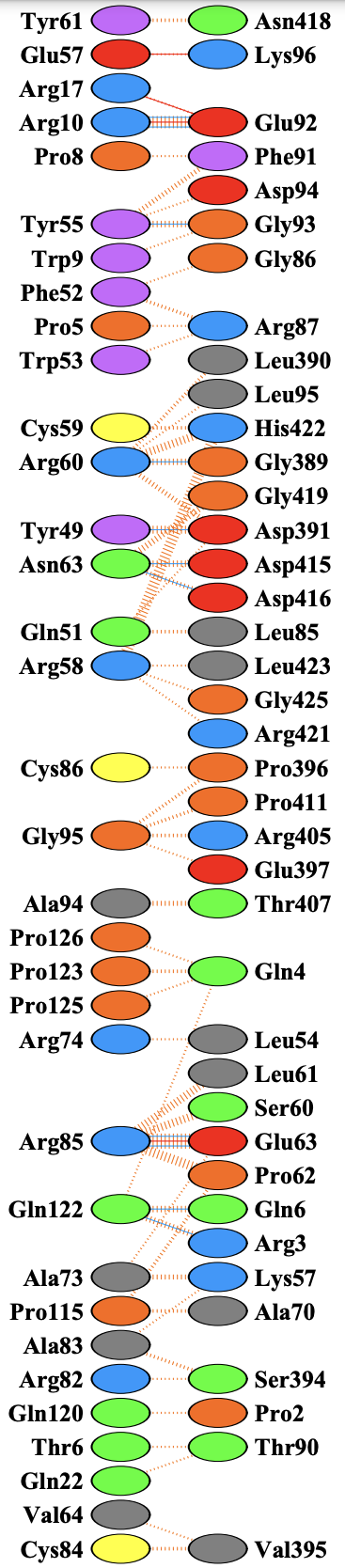


*FasL-MMP9 COVID-19*

List of atom-atom interactions across protein-protein interface

---------------------------------------------------------------

PDB code: jpf3 Chains A }{ B

------------------------------

Hydrogen bonds

--------------

<----- A T O M 1 -----> <----- A T O M 2 ----->

Atom Atom Res Res Atom Atom Res Res

no. name name no. Chain no. name name no. Chain Distance

1. 261 NH2 ARG 17 A <--> 4001 OE2 GLU 92 B 2.81

2. 738 OH TYR 49 A <--> 8427 OD1 ASP 391 B 2.57

3. 848 OH TYR 55 A <--> 4010 O GLY 93 B 2.83

4. 936 NH1 ARG 60 A <--> 8399 O GLY 389 B 3.24

5. 984 ND2 ASN 63 A <--> 8811 O ASP 415 B 2.93

6. 1023 O LEU 65 A <--> 8794 NZ LYS 414 B 2.71

7. 1134 O ALA 73 A <--> 3461 NZ LYS 57 B 2.91

8. 1151 NH1 ARG 74 A <--> 3478 OE1 GLN 58 B 2.59

9. 1285 N CYS 84 A <--> 8469 O SER 394 B 3.08

10. 1294 O CYS 84 A <--> 3510 OG SER 60 B 2.98

11. 1314 NH2 ARG 85 A <--> 3558 OE1 GLU 63 B 2.69

12. 1440 O HIS 93 A <--> 8650 NH1 ARG 405 B 2.87

Non-bonded contacts

-------------------

<----- A T O M 1 -----> <----- A T O M 2 ----->

Atom Atom Res Res Atom Atom Res Res

no. name name no. Chain no. name name no. Chain Distance

1. 1 N VAL 1 A <--> 3211 O MET 41 B 3.15

2. 5 CA VAL 1 A <--> 3211 O MET 41 B 3.53

3. 9 CG1 VAL 1 A <--> 3234 C ARG 42 B 3.86

4. 9 CG1 VAL 1 A <--> 3236 N GLY 43 B 3.68

5. 61 CB PRO 5 A <--> 3909 CZ ARG 87 B 3.77

6. 61 CB PRO 5 A <--> 3910 NH1 ARG 87 B 3.85

7. 64 CG PRO 5 A <--> 3910 NH1 ARG 87 B 3.79

8. 85 O THR 6 A <--> 3959 CB THR 90 B 3.78

9. 85 O THR 6 A <--> 3963 CG2 THR 90 B 3.32

10. 113 CG PRO 8 A <--> 3993 CB GLU 92 B 3.71

11. 113 CG PRO 8 A <--> 3996 CG GLU 92 B 3.88

12. 113 CG PRO 8 A <--> 3999 CD GLU 92 B 3.68

13. 113 CG PRO 8 A <--> 4001 OE2 GLU 92 B 3.41

14. 257 CZ ARG 17 A <--> 4001 OE2 GLU 92 B 3.85

15. 261 NH2 ARG 17 A <--> 3999 CD GLU 92 B 3.16

16. 261 NH2 ARG 17 A <--> 4000 OE1 GLU 92 B 3.09

17. 261 NH2 ARG 17 A <--> 4001 OE2 GLU 92 B 2.81

18. 333 NE2 GLN 22 A <--> 3963 CG2 THR 90 B 3.83

19. 735 CE2 TYR 49 A <--> 8427 OD1 ASP 391 B 3.42

20. 737 CZ TYR 49 A <--> 8427 OD1 ASP 391 B 3.43

21. 738 OH TYR 49 A <--> 8423 CB ASP 391 B 3.77

22. 738 OH TYR 49 A <--> 8426 CG ASP 391 B 3.38

23. 738 OH TYR 49 A <--> 8427 OD1 ASP 391 B 2.57

24. 766 CD GLN 51 A <--> 3881 CD2 LEU 85 B 3.32

25. 767 OE1 GLN 51 A <--> 3881 CD2 LEU 85 B 3.42

26. 768 NE2 GLN 51 A <--> 3867 O ASP 84 B 3.74

27. 768 NE2 GLN 51 A <--> 3881 CD2 LEU 85 B 3.27

28. 787 CE2 PHE 52 A <--> 3867 O ASP 84 B 3.54

29. 789 CZ PHE 52 A <--> 3867 O ASP 84 B 3.37

30. 845 CE2 TYR 55 A <--> 4010 O GLY 93 B 3.33

31. 847 CZ TYR 55 A <--> 4010 O GLY 93 B 3.49

32. 848 OH TYR 55 A <--> 3989 N GLU 92 B 3.89

33. 848 OH TYR 55 A <--> 3991 CA GLU 92 B 3.38

34. 848 OH TYR 55 A <--> 4002 C GLU 92 B 3.43

35. 848 OH TYR 55 A <--> 4004 N GLY 93 B 3.15

36. 848 OH TYR 55 A <--> 4009 C GLY 93 B 3.83

37. 848 OH TYR 55 A <--> 4010 O GLY 93 B 2.83

38. 933 NE ARG 60 A <--> 8930 CD1 LEU 423 B 3.90

39. 935 CZ ARG 60 A <--> 8930 CD1 LEU 423 B 3.63

40. 936 NH1 ARG 60 A <--> 8399 O GLY 389 B 3.24

41. 939 NH2 ARG 60 A <--> 8930 CD1 LEU 423 B 3.57

42. 948 CB TYR 61 A <--> 8913 CD2 HIS 422 B 3.53

43. 948 CB TYR 61 A <--> 8917 NE2 HIS 422 B 3.57

44. 951 CG TYR 61 A <--> 8913 CD2 HIS 422 B 3.75

45. 951 CG TYR 61 A <--> 8917 NE2 HIS 422 B 3.84

46. 954 CD2 TYR 61 A <--> 8913 CD2 HIS 422 B 3.55

47. 958 CE2 TYR 61 A <--> 8848 OD1 ASN 418 B 3.54

48. 988 O ASN 63 A <--> 8811 O ASP 415 B 3.83

49. 988 O ASN 63 A <--> 8808 OD1 ASP 415 B 3.70

50. 979 CB ASN 63 A <--> 8811 O ASP 415 B 3.52

51. 979 CB ASN 63 A <--> 8854 N GLY 419 B 3.88

52. 982 CG ASN 63 A <--> 8811 O ASP 415 B 3.67

53. 984 ND2 ASN 63 A <--> 8455 OG SER 393 B 3.50

54. 984 ND2 ASN 63 A <--> 8811 O ASP 415 B 2.93

55. 991 CA VAL 64 A <--> 8807 CG ASP 415 B 3.89

56. 995 CG1 VAL 64 A <--> 8476 CG1 VAL 395 B 3.68

57. 999 CG2 VAL 64 A <--> 8452 CB SER 393 B 3.86

58. 999 CG2 VAL 64 A <--> 8455 OG SER 393 B 3.80

59. 1005 N LEU 65 A <--> 8807 CG ASP 415 B 3.55

60. 1005 N LEU 65 A <--> 8808 OD1 ASP 415 B 3.16

61. 1005 N LEU 65 A <--> 8809 OD2 ASP 415 B 3.83

62. 1007 CA LEU 65 A <--> 8808 OD1 ASP 415 B 3.88

63. 1022 C LEU 65 A <--> 8794 NZ LYS 414 B 3.79

64. 1023 O LEU 65 A <--> 8791 CE LYS 414 B 3.90

65. 1023 O LEU 65 A <--> 8794 NZ LYS 414 B 2.71

66. 1023 O LEU 65 A <--> 8807 CG ASP 415 B 3.54

67. 1023 O LEU 65 A <--> 8808 OD1 ASP 415 B 3.20

68. 1023 O LEU 65 A <--> 8809 OD2 ASP 415 B 3.17

69. 1133 C ALA 73 A <--> 3455 CD LYS 57 B 3.80

70. 1134 O ALA 73 A <--> 3455 CD LYS 57 B 3.25

71. 1134 O ALA 73 A <--> 3458 CE LYS 57 B 3.11

72. 1134 O ALA 73 A <--> 3461 NZ LYS 57 B 2.91

73. 1150 CZ ARG 74 A <--> 3478 OE1 GLN 58 B 3.42

74. 1151 NH1 ARG 74 A <--> 3477 CD GLN 58 B 3.74

75. 1151 NH1 ARG 74 A <--> 3478 OE1 GLN 58 B 2.59

76. 1154 NH2 ARG 74 A <--> 3478 OE1 GLN 58 B 3.45

77. 1277 CA ALA 83 A <--> 8469 O SER 394 B 3.59

78. 1283 C ALA 83 A <--> 8469 O SER 394 B 3.84

79. 1279 CB ALA 83 A <--> 3466 O LYS 57 B 3.17

80. 1279 CB ALA 83 A <--> 8469 O SER 394 B 3.88

81. 1279 CB ALA 83 A <--> 8463 CB SER 394 B 3.88

82. 1285 N CYS 84 A <--> 8469 O SER 394 B 3.08

83. 1294 O CYS 84 A <--> 3505 CA SER 60 B 3.85

84. 1294 O CYS 84 A <--> 3507 CB SER 60 B 3.35

85. 1294 O CYS 84 A <--> 3510 OG SER 60 B 2.98

86. 1294 O CYS 84 A <--> 8469 O SER 394 B 3.22

87. 1294 O CYS 84 A <--> 8495 CD PRO 396 B 3.87

88. 1292 SG CYS 84 A <--> 8474 CB VAL 395 B 3.46

89. 1292 SG CYS 84 A <--> 8480 CG2 VAL 395 B 3.73

90. 1299 CB ARG 85 A <--> 3513 O SER 60 B 3.79

91. 1302 CG ARG 85 A <--> 3512 C SER 60 B 3.89

92. 1302 CG ARG 85 A <--> 3513 O SER 60 B 3.48

93. 1308 NE ARG 85 A <--> 3558 OE1 GLU 63 B 3.79

94. 1310 CZ ARG 85 A <--> 3534 CA PRO 62 B 3.87

95. 1310 CZ ARG 85 A <--> 3558 OE1 GLU 63 B 3.64

96. 1314 NH2 ARG 85 A <--> 3534 CA PRO 62 B 3.77

97. 1314 NH2 ARG 85 A <--> 3557 CD GLU 63 B 3.61

98. 1314 NH2 ARG 85 A <--> 3558 OE1 GLU 63 B 2.69

99. 1326 SG CYS 86 A <--> 8492 CG PRO 396 B 3.57

100. 1439 C HIS 93 A <--> 8650 NH1 ARG 405 B 3.54

101. 1440 O HIS 93 A <--> 8499 O PRO 396 B 3.16

102. 1440 O HIS 93 A <--> 8649 CZ ARG 405 B 3.59

103. 1440 O HIS 93 A <--> 8650 NH1 ARG 405 B 2.87

104. 1440 O HIS 93 A <--> 8653 NH2 ARG 405 B 3.89

105. 1443 CA ALA 94 A <--> 8649 CZ ARG 405 B 3.67

106. 1443 CA ALA 94 A <--> 8650 NH1 ARG 405 B 3.84

107. 1450 O ALA 94 A <--> 8684 OG1 THR 407 B 3.83

108. 1445 CB ALA 94 A <--> 8677 O PHE 406 B 3.59

109. 1451 N GLY 95 A <--> 8499 O PRO 396 B 3.43

110. 1453 CA GLY 95 A <--> 8499 O PRO 396 B 3.69

111. 1456 C GLY 95 A <--> 8499 O PRO 396 B 3.72

112. 1456 C GLY 95 A <--> 8502 CA GLU 397 B 3.52

113. 1457 O GLY 95 A <--> 8502 CA GLU 397 B 3.73

114. 1457 O GLY 95 A <--> 8514 O GLU 397 B 3.87

115. 1457 O GLY 95 A <--> 8507 CG GLU 397 B 3.51

116. 1458 N PHE 96 A <--> 8499 O PRO 396 B 3.34

117. 1458 N PHE 96 A <--> 8502 CA GLU 397 B 3.61

118. 1485 SG CYS 97 A <--> 8492 CG PRO 396 B 3.69

119. 1720 CB SER 116 A <--> 3527 CD2 LEU 61 B 3.69

120. 1723 OG SER 116 A <--> 3527 CD2 LEU 61 B 3.28

Salt bridges

------------

<----- A T O M 1 -----> <----- A T O M 2 ----->

Atom Atom Res Res Atom Atom Res Res

no. name name no. Chain no. name name no. Chain Distance

1. 261 NH2 ARG 17 A <--> 4001 OE2 GLU 92 B 2.81

2. 1314 NH2 ARG 85 A <--> 3558 OE1 GLU 63 B 2.69

Number of salt bridges: 2

Number of hydrogen bonds: 12

Number of non-bonded contacts: 120

## 1. Ramachandran Plot statistics

**No. of**

**residues %-tage**

**------ ------**

Most favoured regions [A,B,L] 384 77.4%******

Additional allowed regions [a,b,l,p] 104 21.0%

Generously allowed regions [~a,~b,~l,~p] 8 1.6%

Disallowed regions [XX] 0 0.0%

---- ------

Non-glycine and non-proline residues 496 100.0%

End-residues (excl. Gly and Pro) 3

Glycine residues 57

Proline residues 43

----

Total number of residues 599

Based on an analysis of **118** structures of resolution of at least **2.0** Angstroms and R-factor no greater than **20.0** a good quality model would be expected to have over **90%** in the most favoured regions [A,B,L].

## 2. G-Factors

**Average**

**Parameter Score Score**

**--------- ----- -----**

Dihedral angles:-

Phi-psi distribution -0.84*

Chi1-chi2 distribution -0.61*

Chi1 only -0.14

Chi3 & chi4 0.41

Omega  **-1.19****

-0.74*

=====

Main-chain covalent forces:-

Main-chain bond lengths  **-1.33****

Main-chain bond angles  **-2.19****

**-1.83****

=====

OVERALL AVERAGE  **-1.14****

=====

**G-factors** provide a measure of how **unusual**, or out-of-the-ordinary, a property is.

Values below -0.5* - unusual

Values below **-1.0**** - highly unusual

**Important note:** The main-chain bond-lengths and bond angles are compared with the Engh & Huber (1991) ideal values derived from small-molecule data. Therefore, structures refined using different restraints may show apparently large deviations from normality.


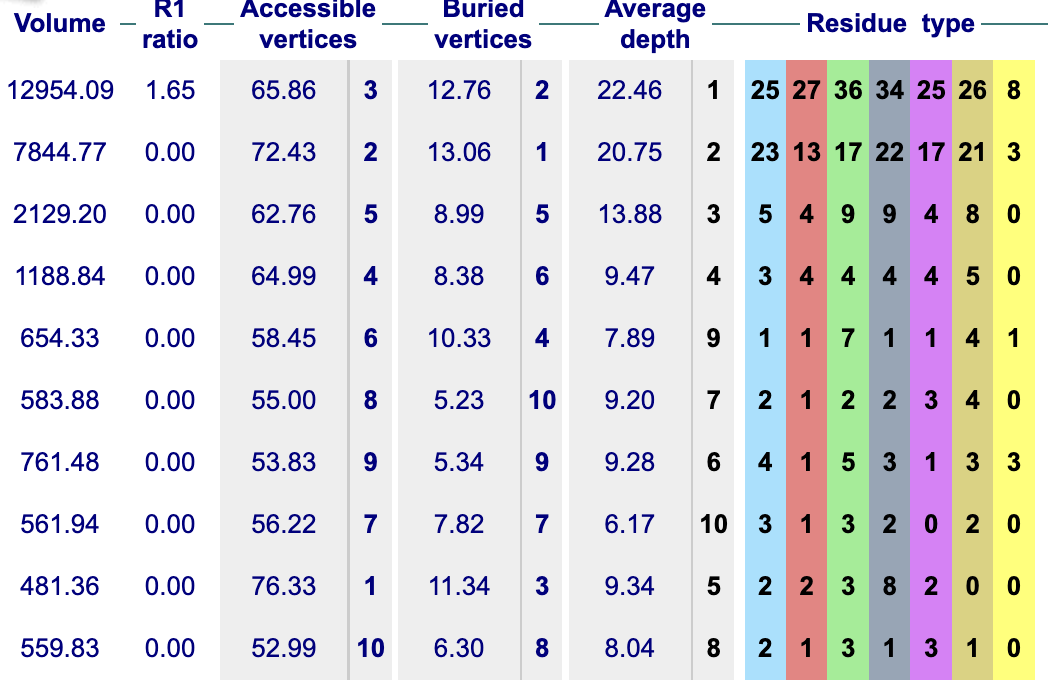


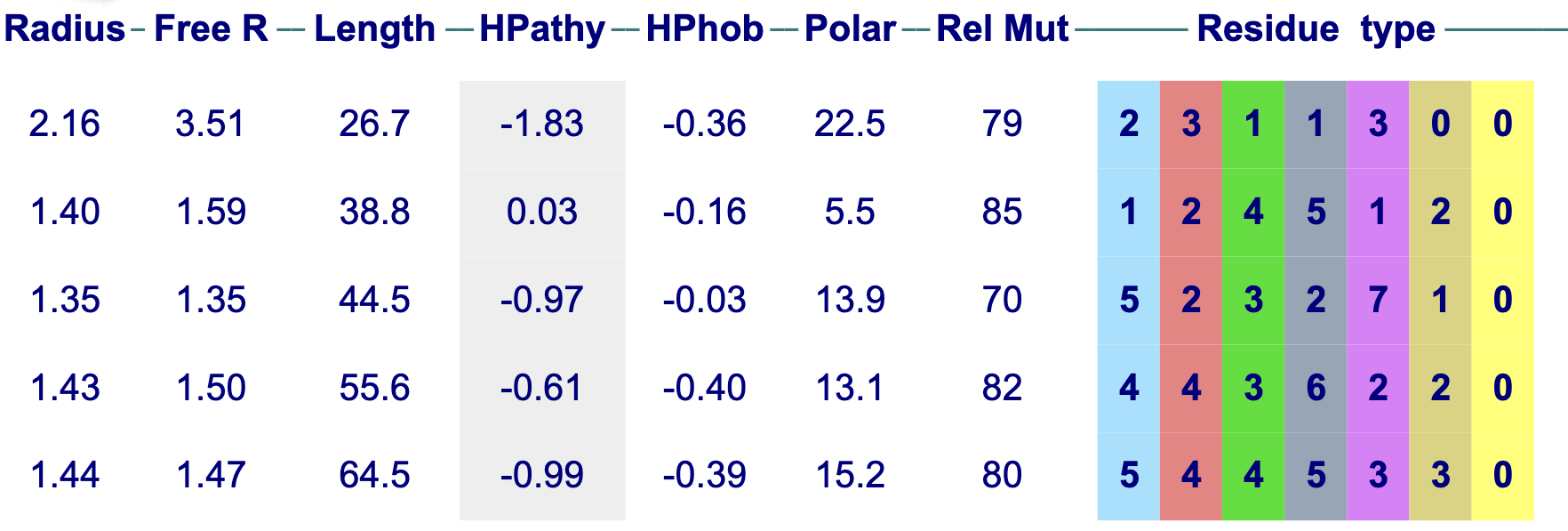

Supplement: Multimedia component 5 [file mmc5.docx]
